# A funnel-type stepwise filtering strategy for identification of potential Q-markers of traditional Chinese medicine formulas

**DOI:** 10.3389/fphar.2023.1143768

**Published:** 2023-05-11

**Authors:** Yuhang Jiang, Mengying Chen, Hongchuan Gang, Xuejiao Li, Chuanjia Zhai, Zhiyang Feng, Gan Luo, Xiaoyan Gao

**Affiliations:** School of Chinese Materia Medica, Beijing University of Chinese Medicine, Beijing, China

**Keywords:** hugan tablets, quality markers, UPLC-Q-exactive-orbitrap/MS, network analysis, characteristic chromatogram, quantitative analysis

## Abstract

Quality marker (Q-marker) serves as an important driver for the standardization of quality control in traditional Chinese medicine (TCM) formulas. However, it is still challenging to discover comprehensive and representative Q-markers. This study aimed to identify Q-markers of Hugan tablet (HGT), a famous TCM formula with ideal clinical effects in liver diseases. Here, we proposed a funnel-type stepwise filtering strategy that integrated secondary metabolites characterization, characteristic chromatogram, quantitative analysis, literature mining, biotransformation rules and network analysis. Firstly, the strategy of “secondary metabolites-botanical drugs-TCM formula” was applied to comprehensively identify the secondary metabolites of HGT. Then, the secondary metabolites with specificity and measurability in each botanical drug were identified by HPLC characteristic chromatogram, biosynthesis pathway and quantitative analysis. Based on literature mining, the effectiveness of botanical metabolites that met the above conditions was evaluated. Furthermore, the metabolism of the above metabolites *in vivo* was studied to reveal their biotransformation forms, which were used for network analysis. At last, according to biotransformation rules of the prototype drugs *in vivo*, the secondary metabolites were traced and preliminarily chosen as Q-markers. As a result, 128 plant secondary metabolites were identified in HGT, and 11 specific plant secondary metabolites were screened out. Then, the content of specific plant secondary metabolites in 15 batches of HGT was determined, which confirmed their measurability. And the results of literature mining showed that eight secondary metabolites had therapeutic effects in treating liver disease at the *in vivo* level, and three secondary metabolites inhibited liver disease-related indicators at the *in vitro* level. After that, 26 compounds absorbed into the blood (11 specific plant metabolites and their 15 metabolites *in vivo*) were detected in rats. Moreover, 14 compounds, including prototype components and their metabolites, were selected as Q-marker candidates by the “TCM formula-botanical drugs-compounds-targets-pathways” network. Finally, 9 plant secondary metabolites were defined as comprehensive and representative Q-markers. Our study not only provides a scientific basis for the improvement and secondary development of the quality standard of HGT, but also proposes a reference method for discovering and identifying Q-markers of TCM preparations.

## Introduction

Quality markers (Q-markers) are chemical components that associated with drug effects (such as effectiveness and safety), and are transferable and traceable components in the process of production and preparation (Liu et al., 2016). The concept of Q-marker integrates medical systems, containing biological properties, manufacturing processes, and formulation theory, to improve the quality and quality control standard of TCM and further promote the relevance of TCM-material basis-quality control effectiveness (Liu, 2017). On this basis, many scholars have systematically conducted Q-markers-related studies from various perspectives. Zhang et al. developed a method for the identification of Q-markers in Chinese herbal medicines by integrating the results of specificity analysis, pharmacokinetic studies, and correlation analysis, using *Corydalis Rhizoma* as an example (Zhang et al., 2016). Zhang et al. also proposed the research approaches of Q-markers of TCM formulas based on “five principles”, and took Shufeng Jiedu capsules as a representative example (Zhang et al., 2018). Chen et al. successfully presented a systemic strategy to discover Q-markers of Shuangshen Pingfei formula for idiopathic pulmonary fibrosis through performing network analysis, pharmacodynamic and pharmacokinetic work (Chen et al., 2022). In conclusion, comprehensive and representative Q-markers of TCM formulas should express multiple attributes simultaneously, including explicit chemical structure, content measurability, being in accord with the theory and practice of TCM, compatibility contribution and specificity (Yang et al., 2017; Xiong et al., 2020).

On this basis, we conclude that the core of Q-markers is effectiveness and representativeness. However, a challenging problem in this domain is how to screen representative and effective Q-markers in TCM preparations composed of a large number of chemical constituents. Network analysis has been widely applied to explore the relationship between botanical drugs, functional targets as well as related diseases from a global perspective due to the advantages of integrity and systematicness (Hao and Xiao, 2014; Zhang et al., 2019; Wang et al., 2021). Whereas, most of the current network analysis studies are based on the ingredients reported in the database or common components *in vitro*, which lack specificity and validity (Zhu et al., 2018; Han et al., 2022; Li et al., 2022). In this study, we established a funnel-type stepwise filtering strategy to identify Q-markers of TCM formulas by comprehensively integrating specificity-measurability-effectiveness-network analysis, so that redundant components could be filtered out.

Hugan tablet (HGT), a Chinese classic formula with definite clinical effect on liver diseases, is derived from Yinchenhao decoction and Xiaochaihu decoction, which has the effects of soothing liver, regulating qi, invigorating spleen as well as dissipating food (Yin et al., 2014; Tang et al., 2018; Yao et al., 2018; Liu et al., 2019; Li X. K. et al., 2021). The prescription of HGT consists of six botanical drugs, monarch medicine that mainly exhibits the effect of soothing liver and relieving depression including Bupleuri Radix (BR, derived from *Bupleurum chinense* DC. or *Bupleurum scorzonerifolium* Willd.); minister medicines, such as Artemisiae Scopariae Herba (ASH, derived from *Artemisia capillaris* Thunb.) and Pulvis Fellis Suis (PFS, derived from *Sus scrofa domestica* Brisson), that exert the efficacy of clearing away dampness and heat, relieving bile and reducing yellowness; assistant medicines, with liver-protecting, heat-clearing and detoxifying properties, containing Schisandrae chinensis Fructus (SCF, derived from *Schisandra chinensis* (Turcz.) Baill.), Isatidis Radix (IR, derived from *Isatis tinctoria* L.) and Mung Bean (MB, derived from *Vigna radiata* L.) R. Wilczek) (Chinese Pharmacopoeia Commission, 2020). Our previous studies confirmed that HGT can effectively alleviate drug-induced liver injury (DILI) induced by atorvastatin through multiple components, targets as well as pathways, and 71 compounds from HGT were identified (Yu and Gao, 2019; Lv et al., 2021). Nevertheless, in terms of quality control of HGT, the 2020 Chinese Pharmacopoeia (ChP) only regulated schisandrin (SSD) as a quality indicator (Chinese Pharmacopoeia Commission, 2020). Studies have shown that SCF can be used to treat liver diseases and has been developed into various pharmaceutical preparations, including SCF liver protection capsules and selenium malt SCF tablets (Yan et al., 2009; Wat et al., 2016; Li Y. et al., 2021). Hence, it seems to lack specificity that merely SSD is employed as a Q-marker for different liver protection formulas. (Li X. K. et al., 2021). Currently, compounds that can represent the overall quality standard of HGT have not been accurately identified. In summary, it is concluded that the HGT is an ideal vehicle to investigate the strategy for the identification of Q-markers of TCM formulas.

**FIGURE 1:**
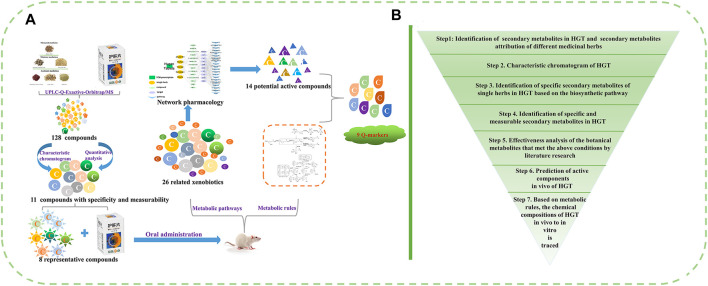
**(A, B)** The funnel-type stepwise filtering strategy for the discovery and identification of potential Q-markers of TCM formulas.

Here, a funnel-type stepwise filtering strategy was proposed and applied to a research example of HGT (Figure 1): 1) The secondary metabolites of HGT and botanical drugs were comprehensively identified by UPLC-Q-Exactive-Orbitrap/MS. 2) The specific secondary metabolites of every botanical drug in HGT were explored via HPLC characteristic chromatogram and biosynthesis pathways. 3) According to the “measurability” of Q-markers, the contents of secondary metabolites in 15 batches of HGT were determined. 4) Based on the “effectiveness” of Q-markers, the effectiveness analysis of the botanical metabolites that met the above conditions was performed by literature research. 5) the biotransformation forms of the special and measurable pharmacological components or active components of HGT that have been identified in the previous research were investigated. 6) On the basis of the above research, the network analysis was used to predict the possible action mechanism and potential Q-markers of HGT. In conclusion, we screened out the Q-markers of HGT progressively based on the attributes of Q-markers, including specificity, measurability and effectiveness. From qualitative to quantitative, from *in vitro* to *in vivo*, a systematic study was conducted to excavate ingredients that could serve as Q-markers. Our study not only provided a new idea about identifying Q-markers in quality control research of TCM formulas, but also screened out reasonable Q-markers of HGT for the first time and improved its quality control level.

## Materials and methods

### Reagents and materials

Reagents and materials are listed in the Supplementary Material.

### Animals, drug administration and serum samples pretreatment

Two-month-old male SD (Sprague-Dawley) rats (200 ± 20 g) were purchased from Beijing Spelford Biotechnology Co., Ltd. (Beijing, China), and were normally raised by the Experimental Animal Center of Beijing University of Chinese Medicine, in a specialized pathogen free (SPF) standard manner. After environment adaptation for 3 days, all rats were randomly grouped and fasted for 12 h before administration with drugs. The studies involving animals were reviewed and approved by the Animal Care and Ethics Committee of Beijing University of Chinese Medicine (approval number: BUCM-4-2022033002-1051).

In the biotransformation profile characterization study, all rats were randomly divided into 10 groups (*n* = 3): the clinical equivalent dose of HGT group, high dose of HGT group and representative compound groups ((R, S)-Goitrin, chlorogenic acid (CA), saikosaponin b2 (SS2), schisandrin (SSD), schizandrol B (SZDB), schisandrin A (SSDA), schisandrin B (SSDB) and schisandrin A (SSDC)). Before administration, all rats were fasted for 12 h and given water freely. On the one hand, the HGT solutions were intragastrically administered to the rats (*n* = 3) at 0.56 g/kg/d (the clinical equivalent dose) and 7.00 g/kg/d (high dose) for 1 day. On the other hand, those representative compounds were orally administered to rats at a dosage of 20 mg/kg/d for 1 day, respectively. Medicated serum was collected from the orbital vein before and after oral administration at 5, 30, 60, 180, 360, 240, 480, 600 as well as 720 min, was centrifuged (4,437 ×g for 10 min at 4°C) and stored at −80°C.

Serum samples were collected at different time points, and were combined to prepare 3 mixed serum samples in every group (A: 5, 30, 60 min; B: 3, 4, 6 h; C: 8, 10, 12 h). Then, the serum sample (1 ml) was treated with methanol (3 mL) and vortexed for 30 s to precipitate the protein. After centrifuging at 14,825 ×g for 15 min, the supernatant was evaporated to dryness under nitrogen gas at room temperature. At last, the residue was redissolved with 100 μl methanol, and the mixtures were vortexed for 30 s and centrifuged at 14,825 *g* for 10 min to obtain the supernatant.

### Preparation of standard solution and sample solution

The HGT consisted of 0.2 g MB, 4.2 g BR, 4.2 g IR, 4.2 g ASH, 0.3 g PFS as well as 2.2 g SCF. The accurately weighed HGT powder (2.0 g, without coating) and 70% methanol (25 ml) were transferred into a 100 mL volumetric flask. Then, the mixtures were ultrasonically extracted for 1 h and centrifuged at 14,825 *g* for 10 min to obtain supernatant. The preparation method of the BR, ASH, SCF, IR, PFS, and MB samples was the same as the above.

Twenty individual standard stock solutions were prepared by dissolving accurately weighed standards in methanol and stored at 4°C in the dark. Then, the mixed standard stock solution for characteristic chromatogram analysis was prepared by employing standard stock solutions of 20 compounds to reach the final concentration (Supplementary Table S1). In the same way, the mixed standard stock solution for quantitative analysis was prepared by using standard stock solutions of 11 specific constituents (Supplementary Table S2).

### Identification of secondary metabolites in HGT

#### Apparatus and UPLC-Q-Exactive-Orbitrap/MS conditions

The UPLC-Q-Exactive-Orbitrap/MS of Thermo Fisher Scientific corporation (Waltham, United States) was used for the identification of the chemical components in HGT and botanical drugs. The chromatography analysis was performed on a Waters ACQUITY UPLC HSS T3 (1.7 μm, 2.1 mm × 100 mm) at 40°C. The mobile phase consisted of 0.1% formic acid in water A) and acetonitrile B). The gradient elution with the following program was carried out: 0–10 min, 2% B–11% B; 10–15 min, 11 B%–20% B; 15–25 min, 20% B–30% B; 25–28 min, 30% B–40% B; 28–47 min, 40% B–98% B; 47–51 min, 98% B; 51–56 min, 98%–2% B; 56–60 min, 2%–2% B. The flow rate was set at 0.3 mL min^-1^ and the injection volume was 2 μl.

After chromatographic separation, electrospray ionization (ESI) was selected to collect MS data in both positive and negative ion modes. The detailed parameter conditions were controlled as follows: the exact mass number was corrected with leucine enkephalin; ion spray voltage in positive ion mode, 3800 V; ion spray voltage in negative ion mode, 3200 V; capillary temperature, 350°C; sheath gas flow rate, 35 Arb; auxiliary gas flow rate, 15 Arb; m/z range, 100–1500 Da.

### Characteristic chromatogram analysis

#### Apparatus and HPLC-PDA conditions

The characteristic chromatogram of HGT was constructed on a UPLC system (Thermo Fisher Scientific, Waltham, United States) equipped with a PDA detector. Chromatographic separation was achieved by a Thermo Syncronis C_18_ column (250 mm × 4.6 mm, 5 μm), and the column temperature was set at 30°C. The mobile phase was composed of 0.1% phosphoric acid aqueous solution A) and acetonitrile B) using a gradient program: 2%–20% B in 0–25 min; 20% B in 25–30 min; 20%–26% B in 30–35 min; 26%–40% B in 35–45 min; 40%–98% B in 45–75 min; 98% B in 75–80 min. The flow rate was controlled at 1 ml min^-1^, and the injection volume was 10 μl. The detection wavelengths were 200 and 250 nm.

#### Method validation

The precision, repeatability and stability experiments were designed, evaluating the stability of the instruments and verifying the reliability of the data. Firstly, a HGT sample was prepared for six times consecutive analyses to assess the precision of the instrument. And the repeatability method was confirmed on account of the continuous analysis of six replicate HGT samples. Furthermore, analyzing one HGT sample at 0, 2, 4, 8, 12 and 24 h was to evaluate the stability of the method, respectively. Finally, to evaluate the method’s durability, chromatographic analysis of a HGT sample was conducted at different column temperatures (33°C, 35°C and 37°C), different flow rates (0.90, 1.00 and 1.10 mL min^-1^) as well as different columns (Thermo Syncronis C_18_, Agilent Zorbax SB-C_18_ and Phenomenex C_18_).

### Quantitative analysis of specific secondary metabolites

#### Apparatus and HPLC-PDA conditions

Chromatographic separation was performed on a Thermo Syncronis C_18_ (250 mm × 4.6 mm, 5 μm), and the column temperature was controlled at 35°C. The 0.3% formic acid in water A) and acetonitrile B) were chosen as the mobile phase. An optimized elution condition was applied as follows: 0–10 min, 2%–7% B; 10–18 min, 7% B; 18–25 min, 7% B–15% B; 25–30 min; 15% B; 30–35 min, 15% B–20% B; 35–40 min, 20% B–26% B; 40–50 min, 26% B–40% B; 50–68 min, 40% B–69% B; 68–72 min, 69% B; 72–86 min, 69% B–98% B; 86–105 min, 98% B. The flow rate was 1 ml min^-1^, and the injection volume was 10 μl. The detection wavelengths were 200 and 250 nm.

#### Method validation

The validation parameters, involving specificity, linearity, precision, stability, reproducibility and recovery of 11 specific components in HGT, were systematically examined. On the basis of the average peak areas of 11 standard solutions at a range of concentrations, the linearities were constructed. And the calibration curves were plotted with the concentration of the homologous standard solution x) as a horizontal coordinate and the average peak area y) as a vertical coordinate. Then, a HGT sample was prepared for 6 times consecutive injections to validate the precision. Six replicate HGT samples in parallel were processed for repeatability evaluation. The sample solution was placed in the UPLC autosampler and analyzed at different periods (0, 2, 4, 8, 12 and 24 h) to assess the stability of the sample. The average recoveries of compounds were investigated by adding a number of standard solutions to 1.0 g HGT powder. Nine samples were prepared parallelly according to the part “Preparation of standard solution and sample solution”. Variations were indicated by percentage relative standard deviations (RSDs).

### Biotransformation rules of specific secondary metabolites

#### Apparatus and UPLC-Q-Exactive-Orbitrap/MS conditions

Chromatographic separation was conducted on a WATERS ACQUITY UPLC HSS T3 (1.7 μm, 2.1 mm × 100 mm) at 40°C. The mobile phase contained 0.1% formic acid aqueous solution A) and acetonitrile B), at a flow rate of 0.3 ml min^-1^. The gradient elution program was applied as follows: 2% B–11% B (0–10 min); 11% B–20% B (10–15 min); 20% B–30% B (15–25 min); 30% B–40% B (25–28 min); 40% B–98% B (28–47 min); 98% B (47–51 min); 98%–2% B (51–51.5 min); 2%–2% B (51.5–55 min). The injection volume was 2 μL.

### Network analysis

First of all, 11 secondary metabolites with specificity and measurability detected in serum and their 15 metabolites (see Supplementary Table S3) were selected as a components pool of HGT, and their targets were collected from the Swiss target prediction (http://www.swisstargetprediction.ch/) (Gao et al., 2022). Two public disease gene-related databases, including GeneCards (https://www.genecards.org/) and MalaCards (Zhao et al., 2022), were applied to retrieve targets for liver diseases. The protein names of these targets were converted into their official gene names via UniProtKB (http://www.uniprot.org/). Then, the overlapping targets of constituents and liver diseases were screened out as potential targets. To investigate their interactions, all potential targets were uploaded into the STRING database. A protein-protein interaction (PPI) network was constructed by Cytoscape 3.7.1 software and topological analysis of nodes in the network was performed (Zhang et al., 2022). GO functional and KEGG pathway enrichment analysis of targets in the PPI network were carried out using DAVID database (https://david.ncifcrf.gov/). In the end, a comprehensive network of “TCM formula-botanical drugs-compounds-targets-pathways” against liver diseases was established by Cytoscape 3.7.1 software.

## Results

### Identification of secondary metabolites in HGT and secondary metabolites attribution of different botanical drugs

Under optimized LC and MS conditions, the UPLC-Q-Exactive Orbitrap/MS technology was adopted to rapidly identify secondary metabolites of HGT as much as possible. However, for the research of TCM formulas, there was a key challenge if the chemical components were identified directly, the high response peaks might cover the small response peaks, resulting in the difficulty of identification. Hence, secondary metabolites of HGT were characterized systematically using the “representative secondary metabolites-botanical drugs-TCM formula” strategy, which was proposed in our previous work (Wang et al., 2017). At first, based on the high-resolution mass spectrometry (HRMS) and literature research, the representative secondary metabolites of various categories in different botanical drugs were analyzed, to sum up corresponding diagnostic ions as well as fragmentation patterns. Then, the secondary metabolites in botanical drugs were certified by the mass spectrometry data processing methods, such as extraction of diagnostic ions, the comparison of MS/MS fragment ions, filtration of neutral loss and so on. Finally, the peaks of TCM formula were compared with the peaks of attested secondary metabolites in botanical drugs, and secondary metabolites in the TCM formula were identified further.

**FIGURE 2:**
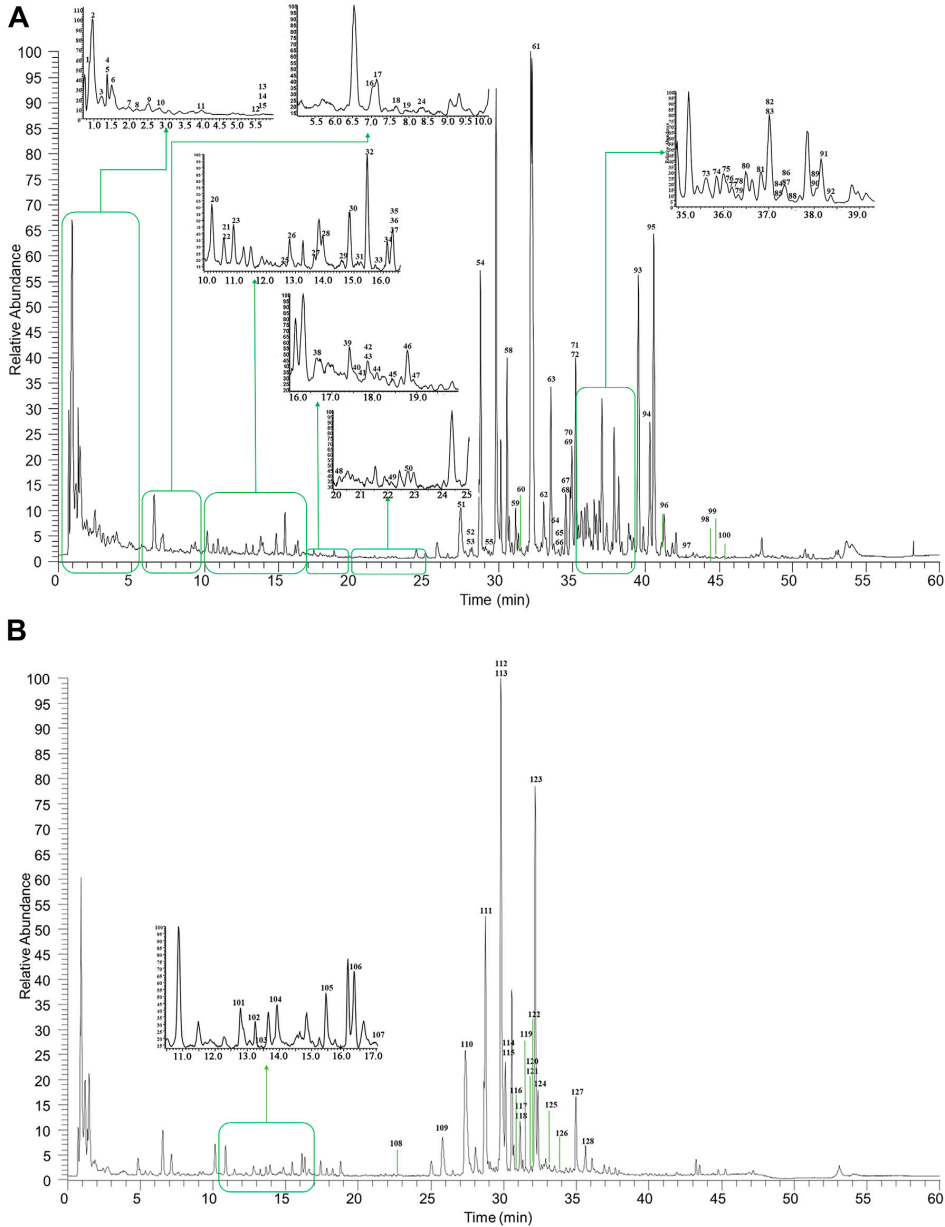
The representative BPCs of HGT in both positive **(A)** and negative **(B)** ion modes.

As illustrated in Figure 2, the base peak chromatograms (BPCs) of HGT were displayed in the positive and negative ion modes. Besides, the total ion chromatograms (TICs) of reference substances and the BPCs of botanical drugs were shown in Supplementary Figures S1–S2. A total of 128 secondary metabolites were identified in HGT, which included 21 flavonoids and their glycosides, 16 triterpene saponins, 10 coumarins, 36 lignans, 19 organic acids, 14 bile acids, 3 nucleic acids as well as 9 other types of compounds. Beyond that, the secondary metabolites attribution of HGT was systematically analyzed, and it was found that 40 secondary metabolites were from SCF, 49 from BR, 14 from PFS, 57 secondary metabolites from ASH, 22 from IR, and 24 from MB. Based on the above results, we identified the exclusive secondary metabolites of different botanical drugs, among which BR had 9 saponins-exclusive metabolites, SCF had 33 exclusive metabolites of dibenzocyclooctene lignans, PFS had 14 exclusive metabolites of bile acids, IR had 2 exclusive metabolites including lignans and alkaloids, and ASH had two exclusive metabolites of flavonoids. The retention time and the MS^n^ data of the identified chemical component in HGT and its botanical drugs were listed in Supplementary Table S4.

### Identification of specific secondary metabolites of botanical drugs in HGT

#### HPLC characteristic chromatogram assay

Under the optimal conditions, the precision, stability, repeatability as well as durability of the method were evaluated with the reference peaks of SS2 (250 nm) and SSDA (200 nm), which contribute to establishing the HPLC characteristic chromatogram. The detailed results were displayed in Supplementary Tables S5–S6 **,** indicating that the established method was feasible and suitable for the quality evaluation of HGT.

**FIGURE 3:**
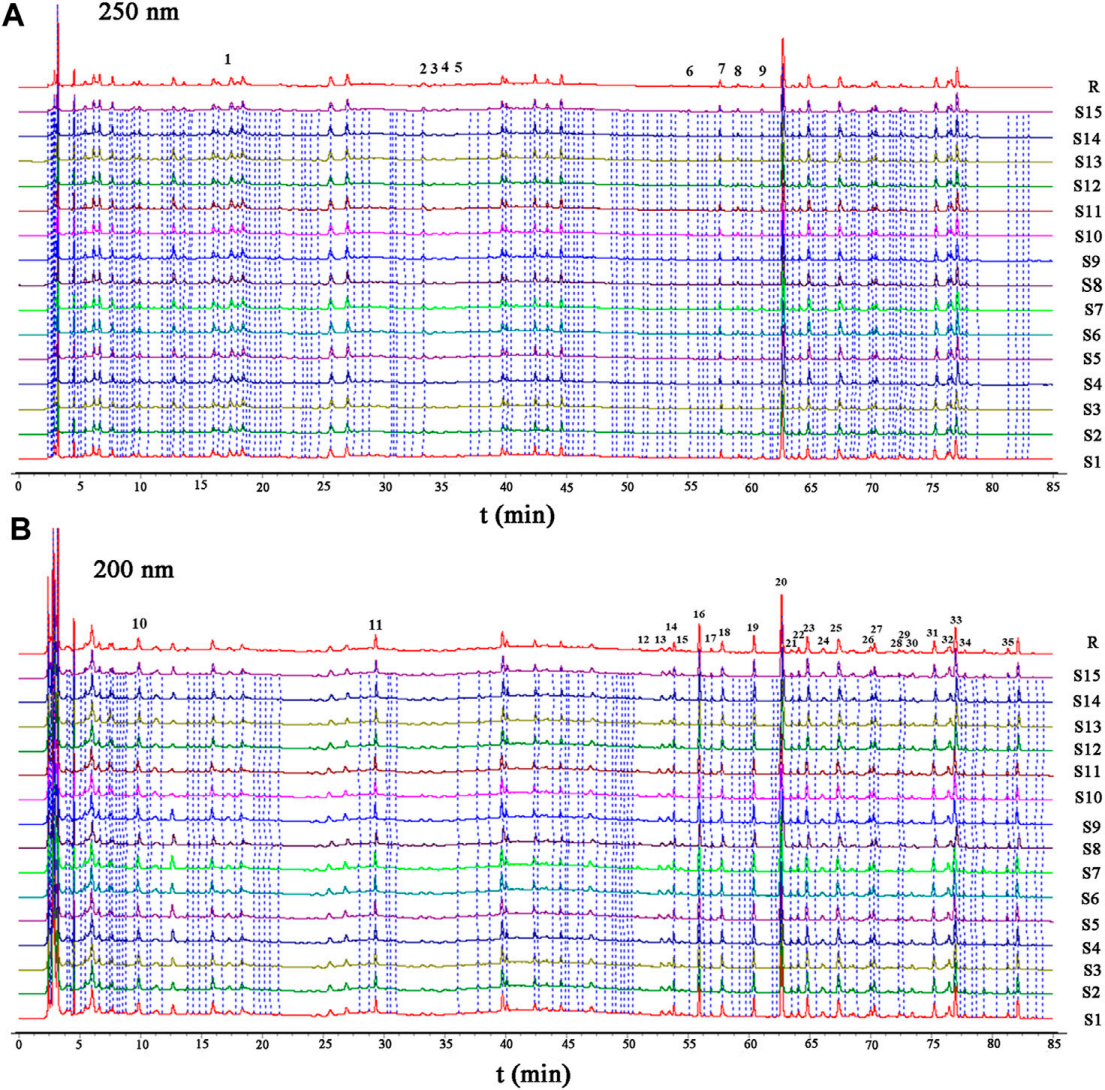
HPLC characteristic chromatogram (**(A)** 250 nm; **(B)** 200 nm) for 15 batches of HGT (S1–S15), and reference chromatogram 1 (R). Note: 1, (R, S)-goitrin (derived from IR); 7, SS2 (derived from BR); 13, TA (derived from PFS); 16, glycochenodeoxycholic acid (derived from PFS); 19, GA (derived from PFS). 20, SSD (derived from SCF), 23, SZDB (derived from SCF), 26, SSTA (derived from SCF), 31, SSDA (derived from SCF), 33, SSDB (derived from SCF), 34, SSDC (derived from SCF).

The validated HPLC method was applied to analyze 15 batches of HGT. The results validated that the similarity of the 15 batches of HGT was greater than 0.97, which conformed to the requirements of ChP. As shown in Figure 3 and Supplementary Figure S3–S4, a total of 35 characteristic peaks were attributed, among which some peaks (NO: 2, 3, 4, and 5) were assigned to ASH, some peaks (NO: 6, 7, 8, and 9) stemmed from BR, some peaks (NO: 1, 10, and 11) owed to IR, some peaks (NO: 20-34) attributed to SCF, and the others (NO: 12-19 and 35) derived from PFS. According to the characterization of secondary metabolites in the above study, the secondary metabolites corresponding to each characteristic peak were analyzed. Finally, as a result of retention behavior, precise molecular weight and fragment ion information, 26 characteristic peaks were identified (IR: 1, ASH: 2, BR: 3, PHS: 8, SCF: 12), of which peaks 1, 7, 13, 16, 19, 20, 23, 26, 31, 33 and 34 were confirmed by the comparison of reference substances **(** Supplementary Table S7 **)**. In conclusion, this study clarified 26 characteristic secondary metabolites, and provided a basis for the discovery of the Q-markers in the following tests.

#### Identification of specific secondary metabolites of botanical drugs in HGT based on the biosynthetic pathway

As shown in Supplementary Figure S5, lignans of SCF are derived from shikimic acid pathway. Based on relevant literature, SSDA is the direct precursor produced from biphenylcycloenoctyl lignans, and is regarded as the common and characteristic biphenylene octyl lignan. Moreover, it has been confirmed that the levels of SSDB, SZDB and schizandrol A derived from SSDA are high in SCF (Wu Z. et al., 2022). The saikosaponins are the main characteristic metabolite of BR, and are synthesized by the mevalonate pathway (Supplementary Figure S6) (Sun et al., 2021; Gu et al., 2022). Meanwhile, SS2 was identified in the HGT characteristic chromatogram (Figure 3 and Supplementary Figure S7). (R, S)-goitrin, a sulfur-containing alkaloid, is the decomposition product of prothyroxine (Supplementary Figure S7). In addition, (R, S)-goitrin is a main metabolite of IR and is used as a quality control indicator in 2020 ChP. CA with hepatoprotective properties is derived from ASH and is considered as one of the major metabolite (Cai et al., 2020). Bile acids, such as taurodeoxycholic acid (TA) and glycohyodeoxycholic acid (GA), are the main active metabolites of PFS, which have anti-inflammatory, liver protection, cholagogic and detoxification effects (Yan et al., 2017; Shi et al., 2018; Chen et al., 2021). Moreover, TA and GA was identified in the HGT characteristic chromatogram described above (Figure 3 and Supplementary Table S5). Therefore, we identified the specific metabolites of each botanical drug in the HGT formulation, and the corresponding results were as follows: SSD, SSDA, SSDB, SSDC, SSTA and SZDB were defined as specific metabolites of SCF, SS2 was a specific metabolite of BR, (R, S)-goitrin was depicted as a specific metabolite of IR, CA was a specific metabolite of ASH and TA was stipulated as a specific metabolite of PFS.

### Quantitative analysis and effectiveness evaluation of specific secondary metabolites

**FIGURE 4:**
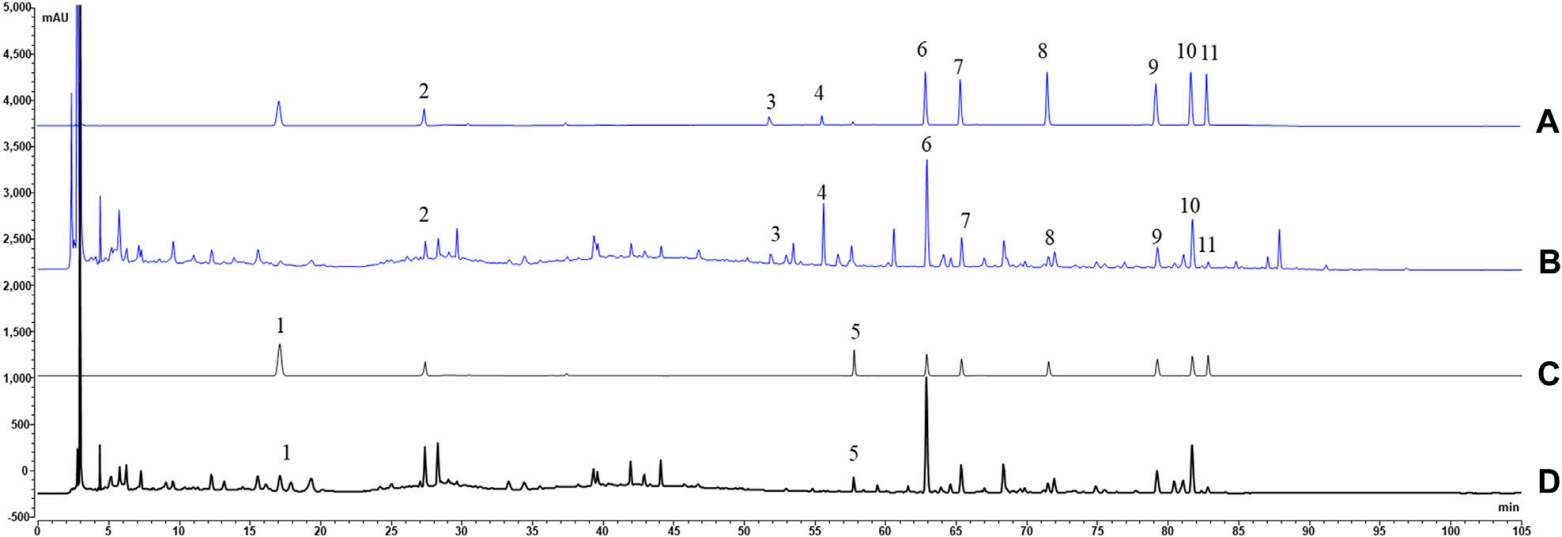
The typical chromatograms of HGT (**(B)** 200 nm; **(D)** 250 nm) and mixed references [**(A)** 200 nm; **(C)** 250 nm]. Note: 1, (R, S)-goitrin (derived from IR); 2, CA (derived from ASH); 3, TA (derived from PFS); 4, GA (derived from PFS); 5, SS2 (derived from BR); 6, SSD (derived from SCF); 7, SZDB (derived from SCF); 8, SSTA (derived from SCF); 9, SSDA (derived from SCF); 10, SSDB (derived from SCF); 11, SSDC (derived from SCF).

In quality control of TCM preparations, the core step is to establish the quantitative analysis method of index components, because the measurability is also a basic requirement of the Q-markers (Lu et al., 2022). Consequently, we developed a quantitative analysis method for specific secondary metabolites to provide data support for the verification of the Q-markers. The typical chromatograms of HGT and mixed standard solution were described in Figure 4. The results demonstrated that the separation degree of 11 index components was ideal and there was no interference in the negative samples, which proved that the specificity of the approach was ideal (Supplementary Figure S8). As depicted in Table 1, 11 specific secondary metabolites showed good linear correlations (*r* values were above 0.9998) in a limited linear range. And the corresponding RSD of precision and stability were less than 2.1% and 2.3%, respectively. Then, the RSD of repeatability variations ranged from 2.2% to 3.8%. Moreover, the recovery of index compounds was proved to be satisfactory as they ranged from 95.10% to 103.99% with RSD of 2.3%–8.1%. Based on the above method, the contents of 11 specific compounds in 15 batches of HGT were calculated, and the results were displayed in Supplementary Table S8. The results showed that the contents of (R, S)-goitrin, CA, TA, GA, SS2, SSD, SZDB, SSTA, SSDA, SSDB and SSDC ranged from 0.021%–0.027%, 0.032%–0.043%, 0.137%–0.189%, 1.097%–1.314%, 0.020–0.024%, 0.155%–0.222%, 0.051%–0.072%, 0.014%–0.019%, 0.042%–0.060%, 0.078%–0.113%, and 0.009%–0.013%, respectively, and the content of the above 11 components fluctuated less between batches.

**TABLE 1:** Linearity curves, precision, stability, repeatability and recovery of 11 specific compounds in HGT.

Compounds	Calibration equation	*r*	Linear range (μg·mL^-1^)	Precision RSD (%)	Stability RSD (%)	Repeatability RSD (%)	Recovery (%)/RSD (%)
(R, S)-goitrin	*y* = 0.5987*x* - 0.8029	1	2.58∼258	1.4	1.6	3.5	100.94/8.1
CA	*y* = 0.3539*x* - 0.1521	1	4.2∼210	1.1	2.3	2.4	95.36/6.2
TA	*y* = 0.086*x* - 0.4178	0.9999	12∼600	1.4	1.8	3.3	95.17/4.6
GA	*y* = 0.0465*x* - 0.0401	0.9999	59.2∼2,960	0.9	1.9	3.4	96.51/5.2
Sb2	*y* = 0.3169*x* + 3.4476	0.9999	0.612∼153	0.8	1.7	2.2	103.68/4.4
SSD	*y* = 0.3191*x* + 2.6889	0.9999	7.16∼1780	0.8	1.8	3.3	95.17/5.0
SZDB	*y* = 0.7911*x* - 2.1893	0.9999	6.6∼330	2.1	2.0	3.5	95.10/3.8
SSTA	*y* = 0.9077*x* - 0.1807	1	0.76∼95	1.0	1.1	3.7	99.85/2.3
SSDA	*y* = 0.6512*x* - 0.0289	0.9999	7.74∼774	1.4	1.6	3.4	101.60/3.7
SSDB	*y* = 0.7291*x* + 0.8044	0.9998	2.9∼290	1.3	1.5	3.5	99.42/5.4
SSDC	*y* = 0.7489*x* + 0.518	0.9999	1.65∼165	1.8	2.2	3.8	103.99/3.9

Based on the above experimental results, 11 plant metabolites in HGT met both the measurability and specificity requirements for Q-markers. In addition, the detailed literature investigation of these 11 plant metabolites was conducted. We found that Schisandra Transaminase reduction capsule with SSD, SSDA and SSDB as the main ingredients has the effects of astringency, regulating qi and transaminase reduction (Ren et al., 2022). And the results of literature mining showed that eight secondary metabolites, including SS2, CA, SSD, SZDB, SSTA, SSDA, SSDB and SSDC, had therapeutic effects in treating liver disease at the *in vivo* level, and three secondary metabolites containing (R, S)-goitrin, TA and GA, inhibited liver disease-related indicator (inflammatory factors) at the *in vitro* level. The detailed literature mining method and results were presented in Supplementary Material and Supplementary Figure S9. In order to quality control of each botanical drug in HGT, we conducted *in vivo* biotransformation studies on these 11 secondary metabolites to obtain their *in vivo* biotransformation forms for network analysis.

### Biotransformation pathways of specific secondary metabolites in rats

After oral administration, TCM formulas, through the synergetic effects of the digestive tract and gastrointestinal flora, produce mixtures consisting of the inherent components and their metabolites, some of which are excreted directly, while others are selectively absorbed and enter the bloodstream under the assistance of liver metabolizing enzymes to exert medicinal effects (Li et al., 2018). Admittedly, only the ingredients absorbed into the blood are likely to be considered as active ingredients. Moreover, the peaks of some metabolites in serum cannot be examined due to the weak mass spectrometric response and the lack of MS/MS information. According to the results of prototypes *in vivo*, eight secondary metabolites were screened out as the representative secondary metabolites of HGT to investigate their typical biotransformation pathways *in vivo*, which contributed to the biotransformation profile analysis *in vivo* of HGT as a complicated system. Therefore, a new strategy of “from monomer to TCM formula, from high dose to clinical equivalent dose” was introduced to identify effective ingredients *in vivo* of TCM formula and provide chemical compounds basis for the next network analysis.

**FIGURE 5:**
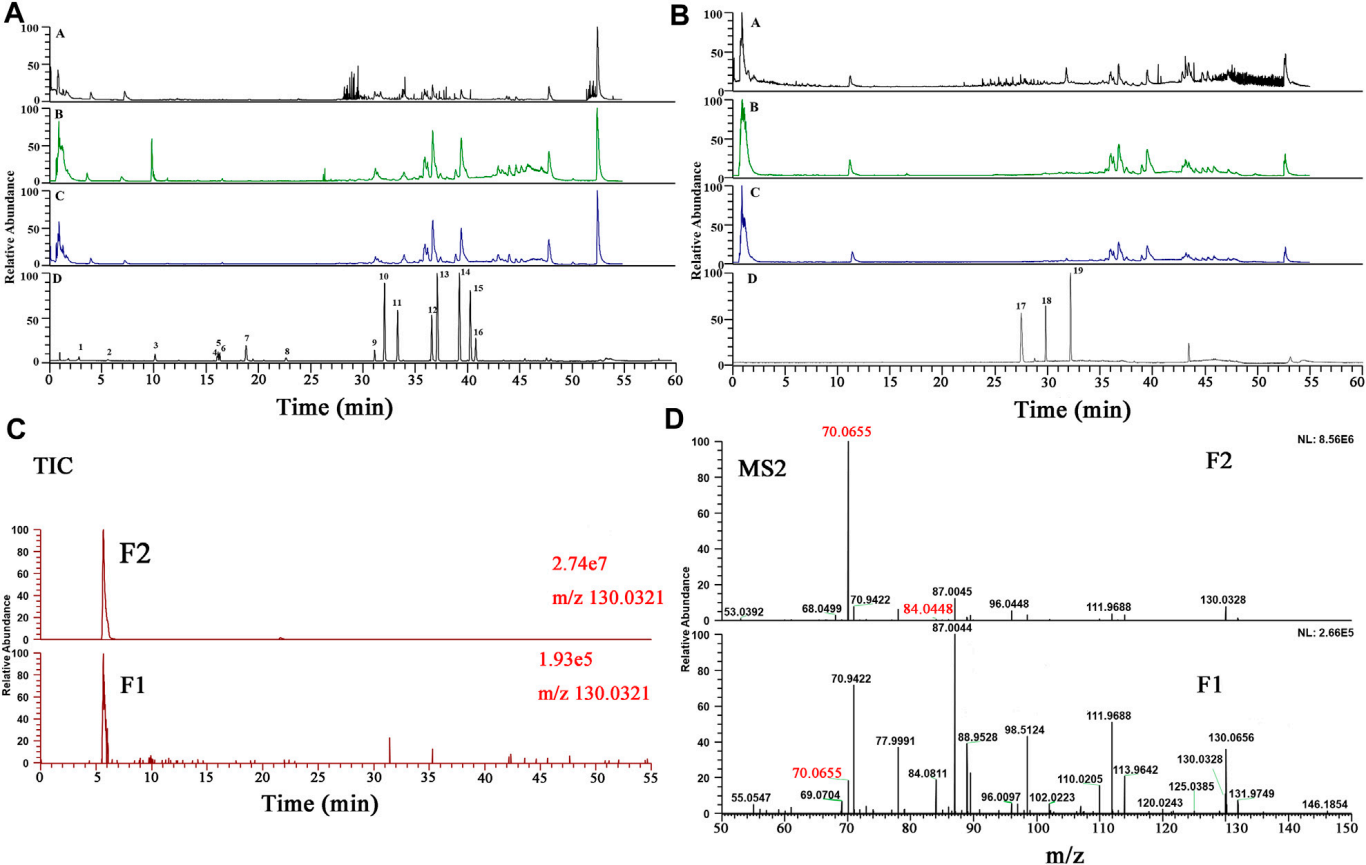
**(A, B)**: BPCs of different samples in both positive and negative ion modes (A: blank serum sample; **(B)** serum sample from high dose HGT group; **(C)** serum sample from clinically equivalent dose HGT group; **(D)** Mixed reference substances); **(C, D)**: The TICs and MS^2^ spectrum of F1 and F2.

**FIGURE 6:**
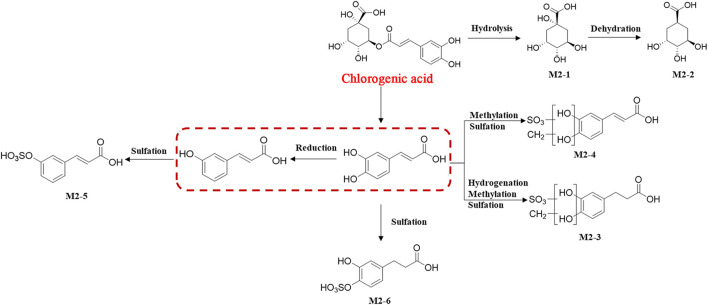
The schematic diagram of possible biotransformation pathways of CA.

**FIGURE 7:**
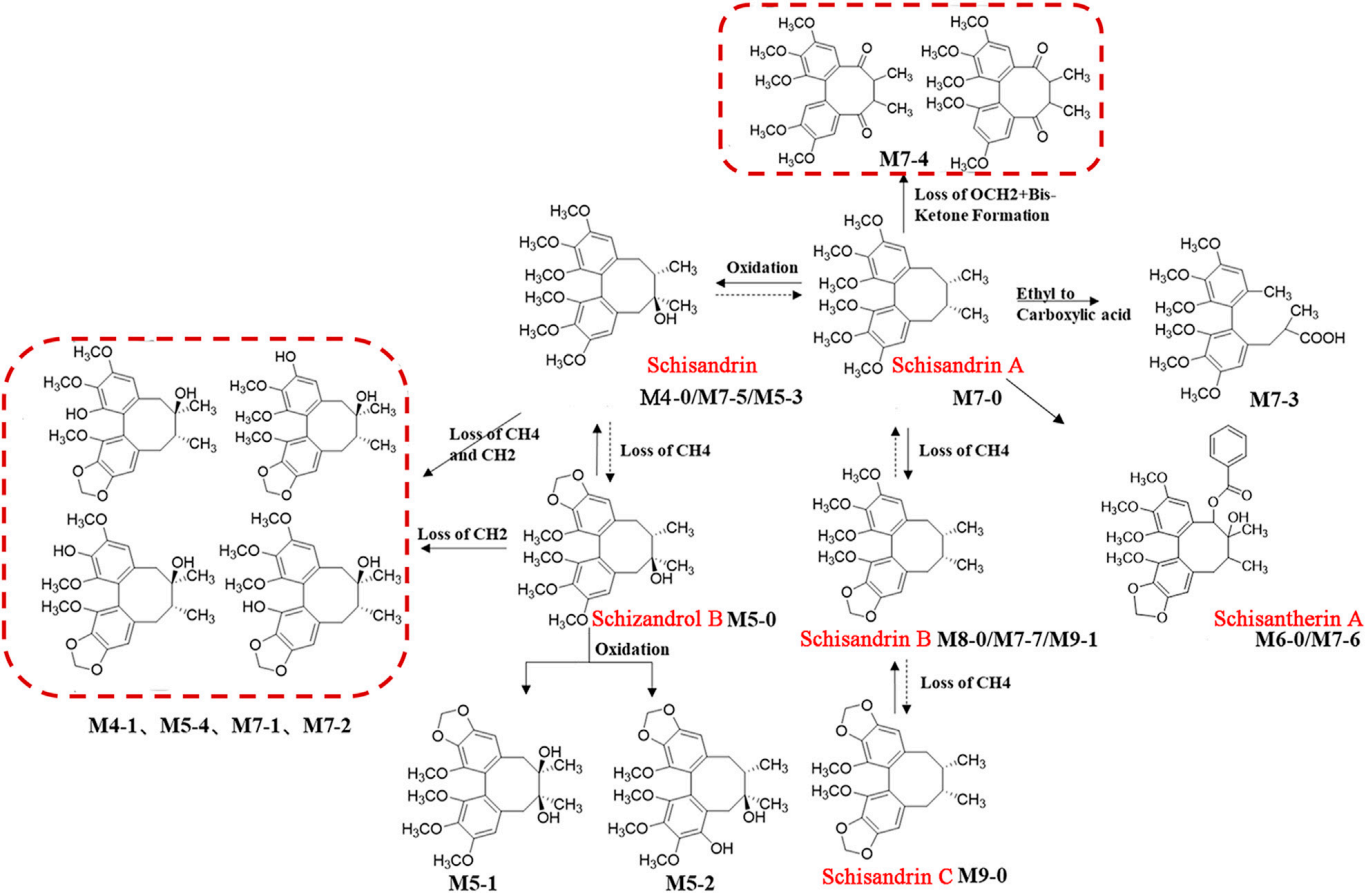
The schematic diagram of possible biotransformation pathways of lignans. Note: Solid lines indicate biotransformation pathways detected in serum; the dashed lines indicate the biotransformation pathways hypothesized from the literature.

Based on the strategy of “from monomer to TCM formula, from high dose to clinical equivalent dose” strategy, 11 prototypical secondary metabolites and 15 metabolites derived from HGT were identified in rats after oral administration of clinical equivalent dose groups (Figures 5A,B), suggesting that these compounds were screened out as blood-entry components of HGT. The MS^n^ data of 26 components were summarized in Supplementary Table S9. CA has six metabolites in rats and is involved in a variety of biotransformation reactions such as hydrolysis, methylation, glucuronidation, sulfation, and dehydration. The schematic diagram of biotransformation pathways was shown in Figure 6. Lignans mainly undergo phase I biotransformation transformation *in vivo*, namely, oxidation, demethylation, methylation, methylenedioxy ring opening as well as hydroxylation, whose biotransformation pathway was displayed in Figure 7.

Taking the identification of a metabolite as an example, the F2 peak with a higher response (2.74e^7^) was observed in the extracted ion chromatogram (EIC) of a representative compound group (Figure 5C). In contrast, at the same retention time, the response of F1 was lower (1.93e^5^) in the clinical equivalent dose of HGT group, leading to its fragment ions with small response values were disturbed by other fragment ions (Figure 5D). Firstly, F2 and F1 could be regarded as identical compound because of their same retention time. And according to corresponding MS^n^ data, observing the precursor ion of [M + H]^+^ of F2 at m/z 130.0323, the fragment ions m/z 70.0655 as well as m/z 84.0448, which were characteristic fragment ions. Finally, F2 was identified as (R, S)-goitrin by the comparison with MS^n^ data and retention time of the reference standard. Taken together, our proposed method can not only effectively identify low-response metabolites, but also enhance the reliability of metabolite identification in serum.

### Identification of Q-markers based on network analysis and biotransformation rules

**FIGURE 8:**
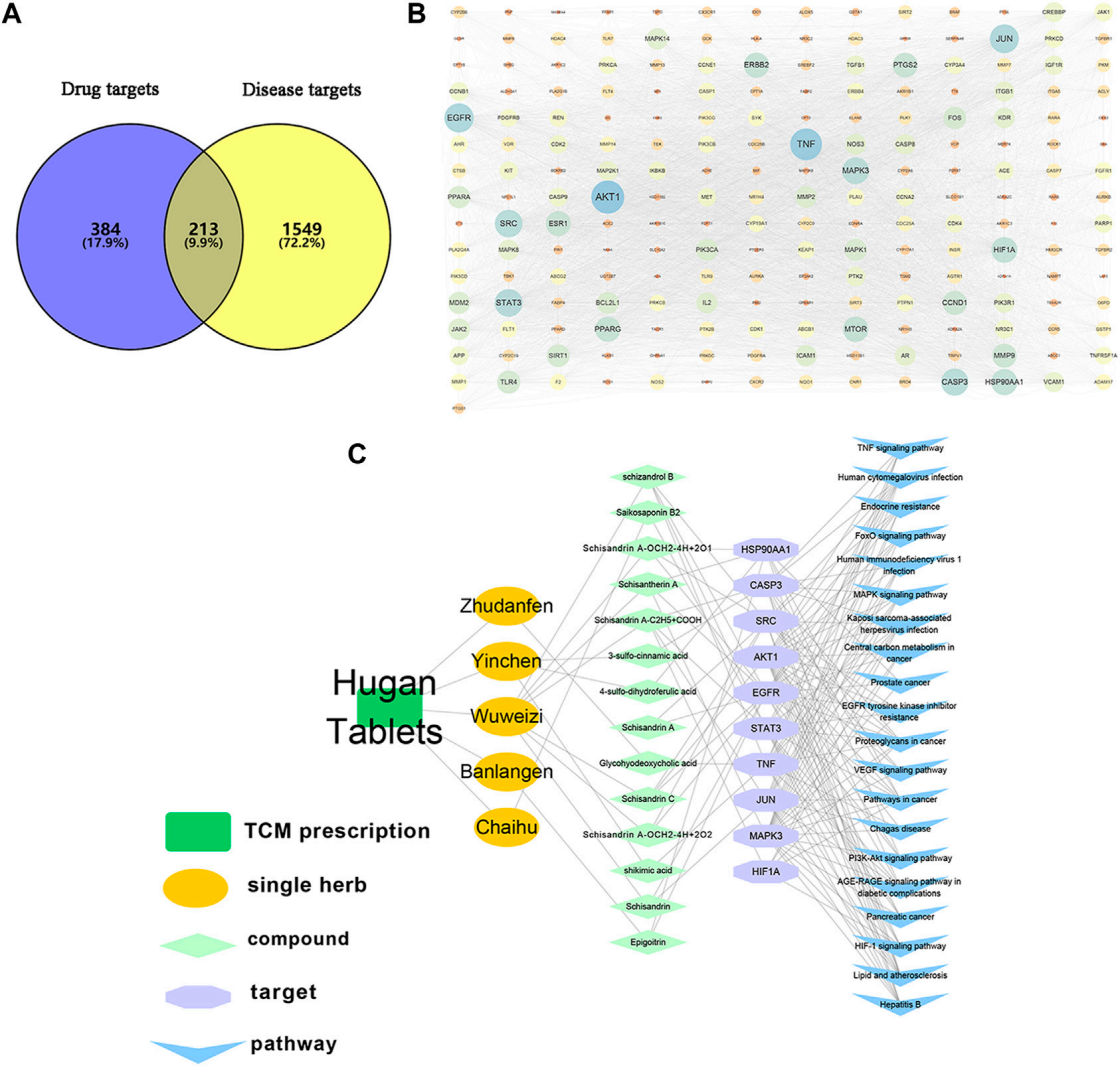
Network pharmacology analysis of HGT in the treatment of liver diseases. **(A)** common targets of HGT and liver diseases. **(B)** PPI network of potential targets. **(C)** network diagram of “TCM formula-single herb-compounds-targets-pathways”.

A comprehensive network of “TCM formula-botanical drugs-components-targets-pathways” was built to reveal the complex interactions, and screen out Q-marker candidates of HGT. Making use of the corresponding database, 597 potential targets of 26 components in serum were obtained, and 1762 liver diseases-related human targets were collected. Subsequently, the targets of the compounds intersected with the disease targets to form 213 overlapping targets (Figure 8A), and a PPI network was established based on their interactions (Figure 8B). Furthermore, the top 10 targets ranked by degree value (AKT1, TNF, EGFR, JUN, STAT3, SRC, CASP3, MAPK3, HSP90AA1 and HIF1A) were selected as crucial targets of HGT for liver diseases (Table 2). To investigate the therapeutic mechanisms of HGT in the treatment of liver diseases, the KEGG pathway enrichment analysis was carried out. Accordingly, 165 related pathways were collected, and the top 20 pathways with the smallest *p*-value were deemed as the main pathways (Supplementary Table S10), including signal transduction pathways, cancer-related pathways, immune signaling pathways as well as liver diseases-related pathways. At last, a “TCM formula-botanical drugs-components-targets-pathways” map was constructed (Figure 8C), which contained HGT formula, 5 botanical drugs, 14 Q-marker candidates, 10 key targets and 20 main pathways. This network suggested that HGT exerted curative effect through the action of multiple components and multiple targets, and these 14 Q-marker candidates might be the key active ingredients. According to biotransformation rule in the above study, we retraced the prototype composition of 14 compounds, among which shikimic acid, 3-sulfo-cinnamic acid and 4-sulfo-dihydroferulic acid were derived from CA; SSDA-C_2_H_5_+COOH and SSDA-OCH_2_-4H+2O from SSDA. Other compounds, containing SZDB, SS2, SSD, (R, S)-goitrin, SSTA, SSDC, SSDA and GA, could exert their effects directly *in vivo* in the form of prototype components. Therefore, integrating network analysis and biotransformation pathway research of specific secondary metabolites, 9 secondary metabolites were identified as Q-markers of HGT: SS2 in BR, (R, S)-goitrin in IR, CA in ASH, SSD, SZDB, SSTA, SSDA, SSDC in SCF and GA in PFS.

**TABLE 2:** 10 key targets of HGT in the treatment of liver diseases.

Gene symbol	Uniprot ID	Description	Degree
AKT1	P31749	Serine/threonine-protein kinase AKT	139
TNF	P01375	TNF-alpha	131
EGFR	P00533	Epidermal growth factor receptor erbB1	114
JUN	P05412	Proto-oncogene c-JUN	111
STAT3	P40763	Signal transducer and activator of transcription 3	105
SRC	P12931	Tyrosine-protein kinase SRC	103
CASP3	P42574	Caspase-3	101
MAPK3	P27361	MAP kinase ERK1	99
HSP90AA1	P07900	Heat shock protein HSP 90-alpha	93
HIF1A	Q16665	Hypoxia-inducible factor 1 alpha	92

## Discussion and conclusion

Here, a funnel-type stepwise filtering strategy integrating secondary metabolites characterization, characteristic chromatogram, quantitative analysis, literature mining, biotransformation pathways and network analysis was successfully developed and applied to screen out 9 representative Q-markers of HGT. Admittedly, we currently only speculated that these 9 Q-markers might be active ingredients of HGT through network analysis. However, as mentioned above, relevant studies reported that 9 Q-markers had therapeutic effects on liver disease at the *in vivo* or *in vitro* levels, which provided clear literature support for our study and demonstrates the reliability of our research results to a certain extent. In view of the above views, 9 Q-markers of HGT identified by the funnel-type stepwise filtering strategy possess comprehensive features of compatibility, quality transitivity and traceability, specificity, measurability and effectiveness to some extent. Whereas, there are some limitations in this study, such as the effectiveness of Q-markers has yet to be confirmed on the basis of *in vivo* and *in vitro* experiments.

Noteworthy, our study not only systematically identified Q-markers of HGT, but also conducted an exploratory study on the mechanism for the treatment of liver diseases based on network analysis. Based on the integrated network, 9 Q-markers may regulate phosphatidylinositol-3-kinase/Akt (PI3K-Akt), FoxO, tumor necrosis factor (TNF), mitogen-activated protein kinase (MAPK), HIF-1, vascular endothelial growth factor (VEGF) and other signaling pathways through acting on AKT1, TNF, EGFR, JUN, STAT3, SRC, CASP3, MAPK3, HSP90AA1 and HIF1A targets to achieve the protective effect on the liver. Oxidative stress has been closely associated with almost all human liver diseases (Kaur et al., 2022). The related research has reported that the FoxO signaling pathway is involved in oxidative stress-induced apoptosis and plays a significant role in regulating the cellular oxidative stress response. In addition, FoxO3 can promote apoptosis of hepatocytes under oxidative stress (Tao et al., 2013; An et al., 2020). Therefore, HGT may reduce hepatocyte apoptosis under oxidative stress by acting on AKT1, EGFR, STATS and MAPK3 targets to modulate the FoxO signaling pathway. Besides, it has been proved that HSCs proliferation is promoted by activating the PI3K-Akt signaling pathway, which accelerates the occurrence and progression of liver fibrosis (Kao et al., 2017; Wu X. et al., 2022). These results suggest that HGT may inhibit the activation of the PI3K-Akt signaling pathway through CASP3, AKT1, EGFR and MAPK3, thereby inhibiting the activation of HSC and the occurrence of liver fibrosis. The development of liver diseases is usually accompanied by an inflammatory response (Kubes and Jenne, 2018), and it has been proved that the unbalanced TNF-α signaling pathway can activate an inflammatory cascade response, triggering excessive cytokine/chemokine release and cell death (Lai et al., 2019). Hence, it is speculated that HGT may regulate TNF signaling pathway through AKT1, TNF, MAPK3, CASP3 and JUN, thus inhibiting inflammation and protecting hepatocytes from apoptosis. MAPK signaling pathway plays a crucial role in mediating cellular oxidative stress, proliferation as well as apoptosis, which is associated with liver-related inflammatory response, liver fibrosis and other pathological activities (Gui et al., 2019). And it was demonstrated that acute liver injury (ALI) may be related to the MAPK signaling pathway closely, and effective inhibition of the MAPK signaling pathway may be essential for liver protection (Li Y. et al., 2021). VEGF, a key angiogenesis-promoting factor, plays a significant role in the regulation of different types of angiogenesis (Zheng et al., 2021). In addition, it was illustrated that VEGF is associated with the occurrence and development of liver fibrosis (Deng et al., 2019). Thus, HGT may regulate the VEGF signaling pathway by regulating the expression of AKT1, SRC and MAPK3, preventing further aggravation of hepatic sinusoidal capillarization and delaying the process of liver fibrosis.

## Figures and Tables

**Table:** 

**APAP**	Acetaminophen
**ASH**	Artemisiae Scopariae Herba
**BPC**	base peak chromatogram
**BR**	Bupleuri Radix
**CA**	chlorogenic acid
**ChP**	Chinese Pharmacopoeia
**DAVID**	The Database for 19 Annotation, Visualization and Integrated Discovery
**EIC**	extract ion chromatogram
**ESI**	electrospray ionization
**GA**	glycohyodeoxycholic acid
**HGT**	Hugan tablets
**HPLC**	High Performance Liquid Chromatography
**HRMS**	high-resolution mass spectrometry
**HSC**	hepatocyte stellate cells
**IR**	Isatidis Radix
**MAPK**	mitogen-activated protein kinase
**MB**	Mung Bean
**NF-κB**	nuclear factor‐κB
**PFS**	Pulvis Fellis Suis
**PI3K-Akt**	phosphatidylinositol-3-kinase/Akt
**PPI**	protein-protein interaction
**Q-marker**	quality marker
**SSD**	schisandrin
**SS2**	saikosaponin b2
**SD**	Sprague-Dawley
**SCF**	Schisandrae chinensis Fructus
**SPF**	specialized pathogen free
**SSDA**	schisandrin A
**SSDB**	schisandrin B
**SSDC**	schisandrin C
**SZDB**	schizandrol B
**TA**	taurodeoxycholic acid
**TCM**	traditional Chinese medicine
**TIC**	total ion chromatograms
**TNF**	tumor necrosis factor
**UPLC-Q-Exactive-Orbitrap/MS**	Ultra performance liquid chromatography-quadrupole-exactive orbitrap mass
**VEGF**	vascular endothelial growth factor

## Data Availability

The original contributions presented in the study are included in the article/Supplementary Material. Further inquiries can be directed to the corresponding author.
