# Supplementary material for: A funnel-type stepwise filtering strategy for identification of potential Q-markers of traditional Chinese medicine formulas
Source: Front Pharmacol. 2023 May 11;14:1143768. doi: 10.3389/fphar.2023.1143768 (PMC10213786; doi:10.3389/fphar.2023.1143768)
Supplement: Supplementary file 1 [file Table1.doc]

Supplementary Material

**Supplementary Figures and Tables**

**Supplementary Figures**


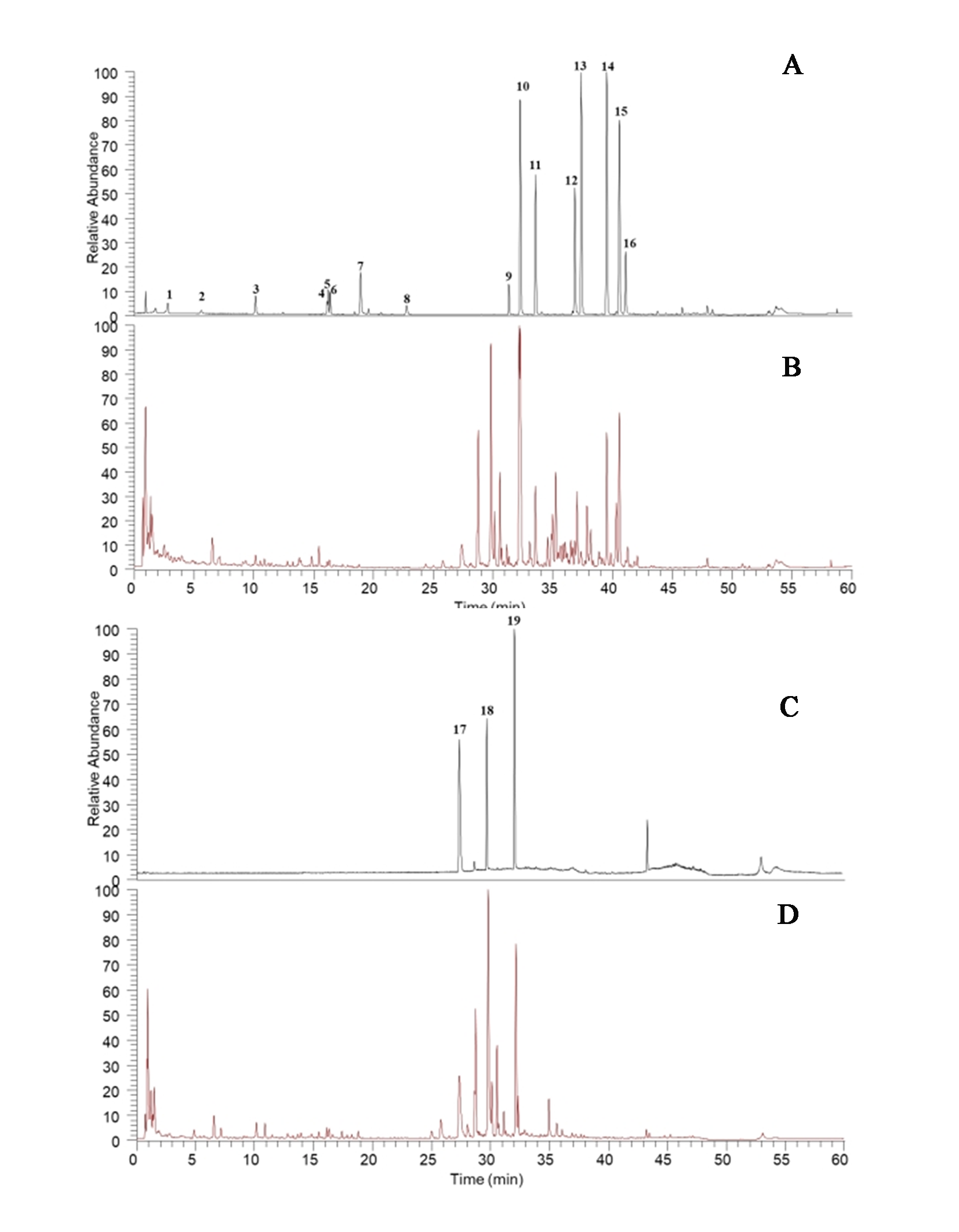


**Supplementary Figure 1.** TICs of Hugan tablets and mixed reference substances in both positive **(A and B)** and negative **(C and D)** ion modes.


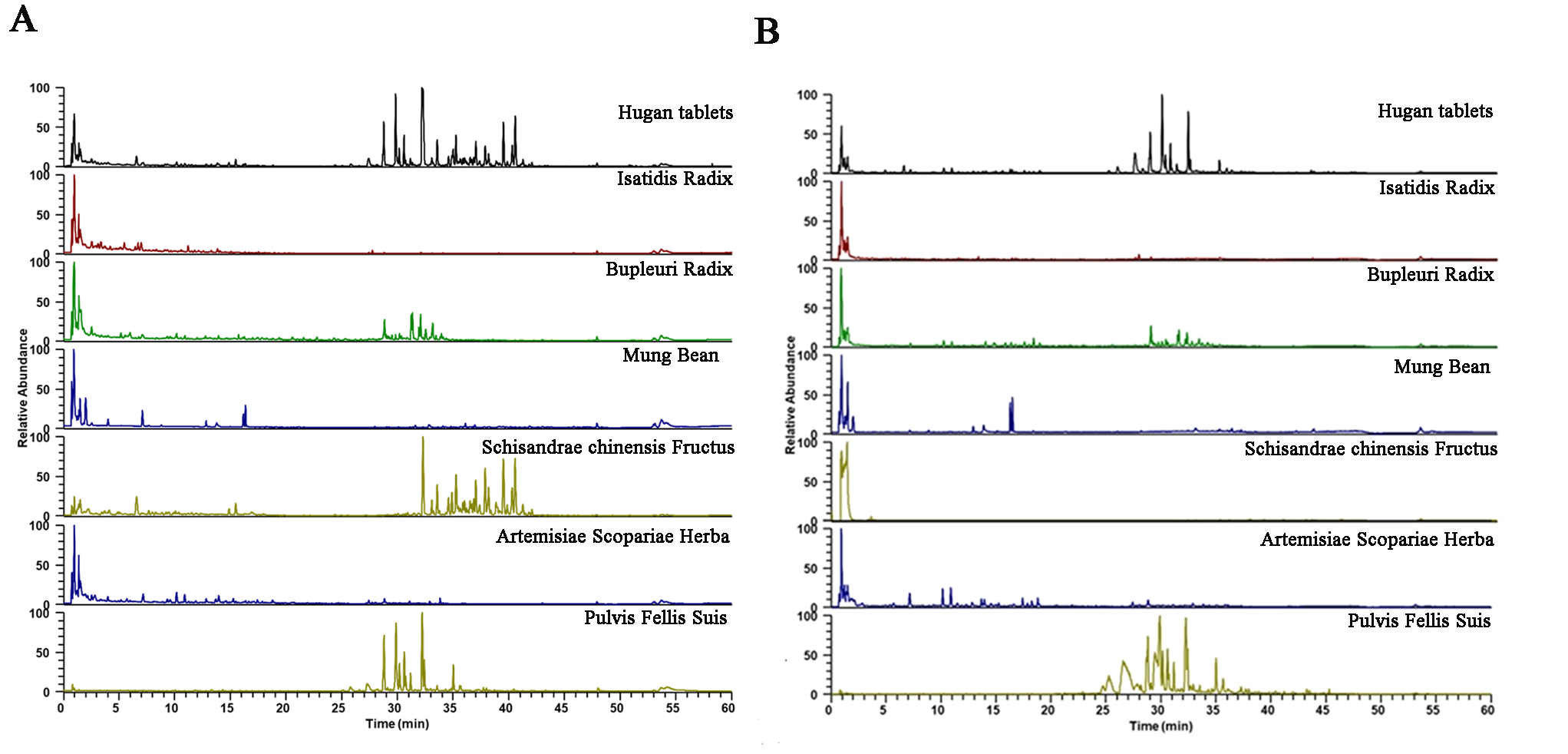


**Supplementary Figure 2.** BPCs of Hugan tablet and single herb in both positive **(A)** and negative **(B)** ion modes.


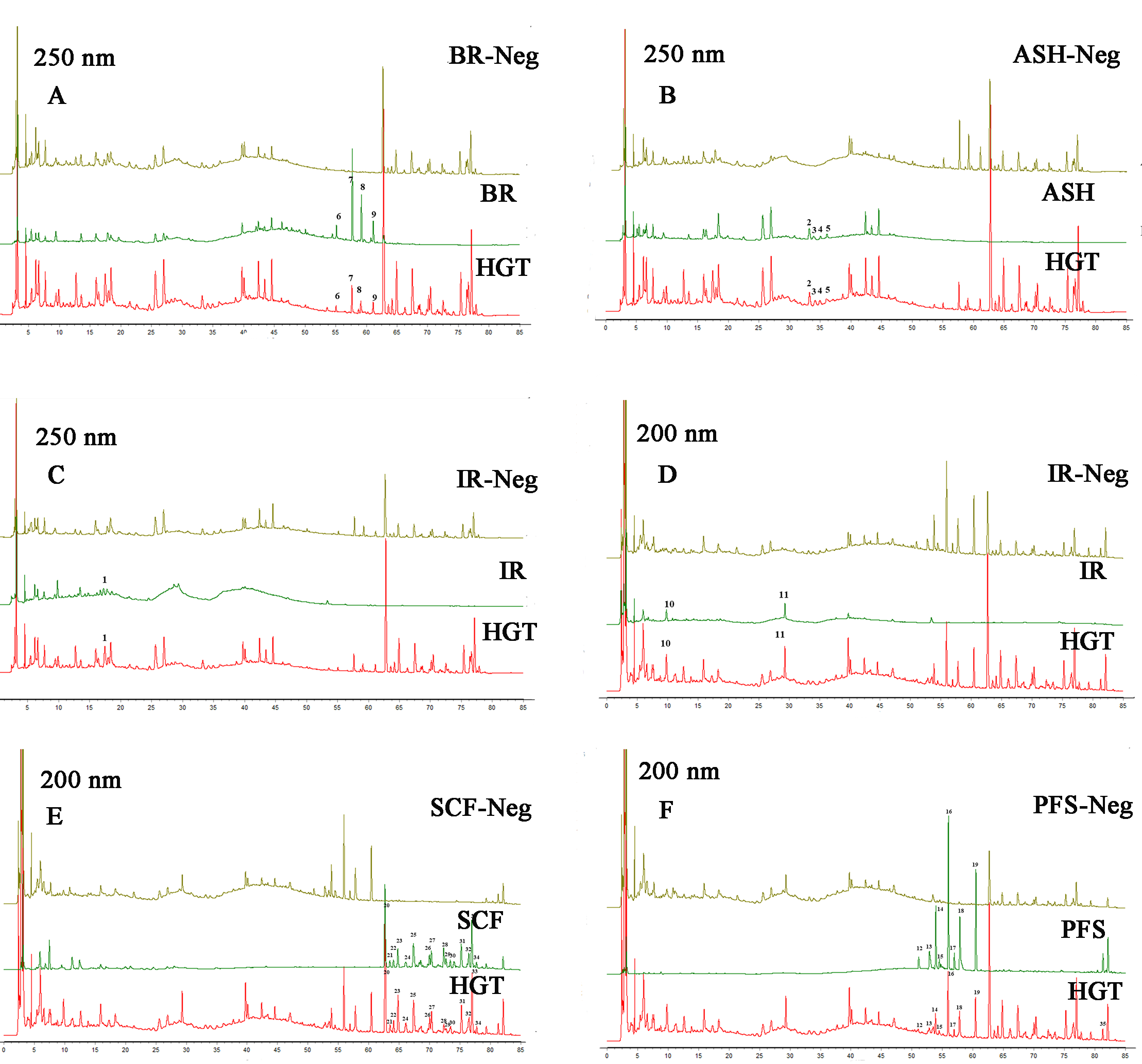


**Supplementary Figure 3.** The characteristic peaks of each herb in HGT.


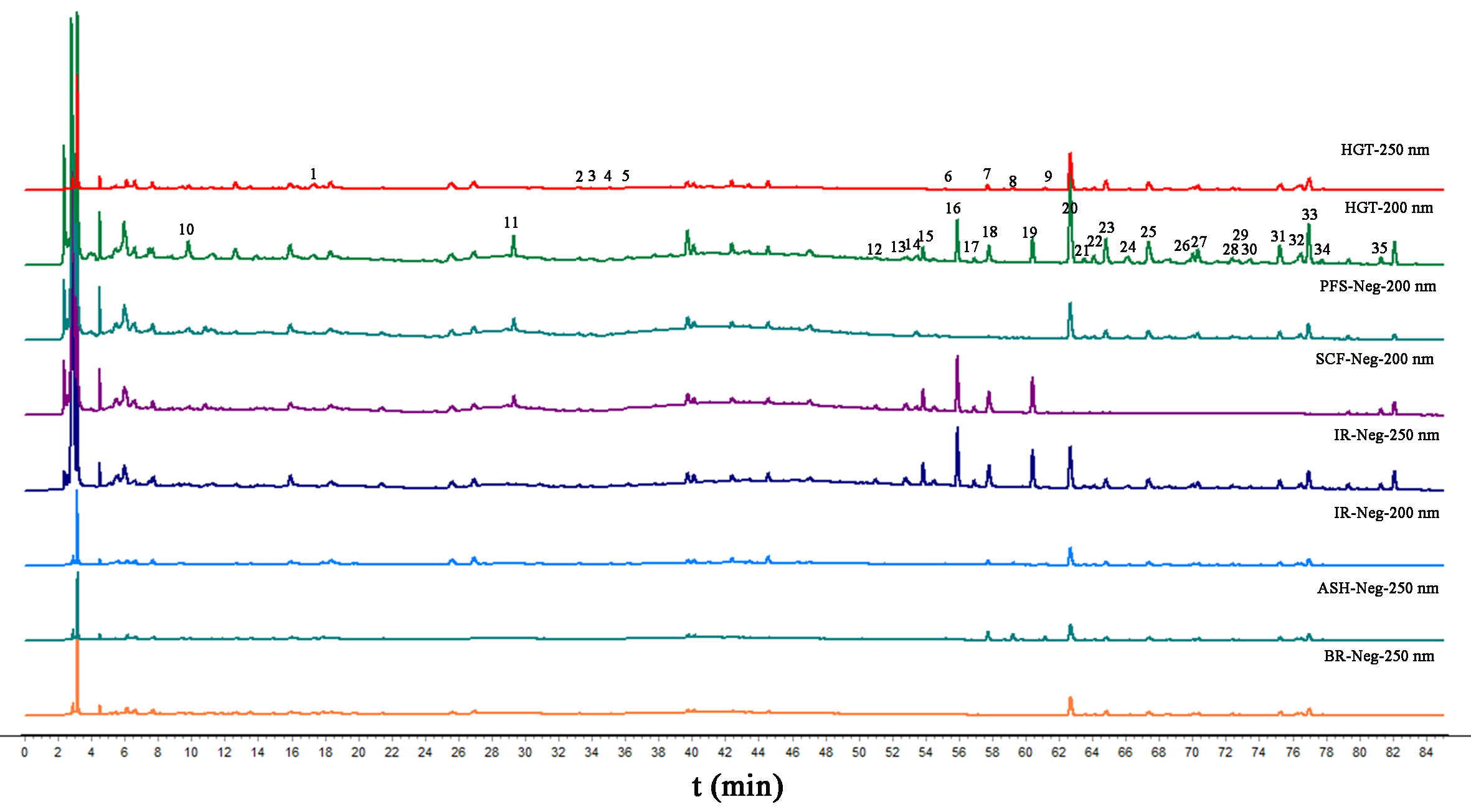


**Supplementary Figure 4.** 35 characteristic peaks in HGT characteristic chromatogram and their attribution.


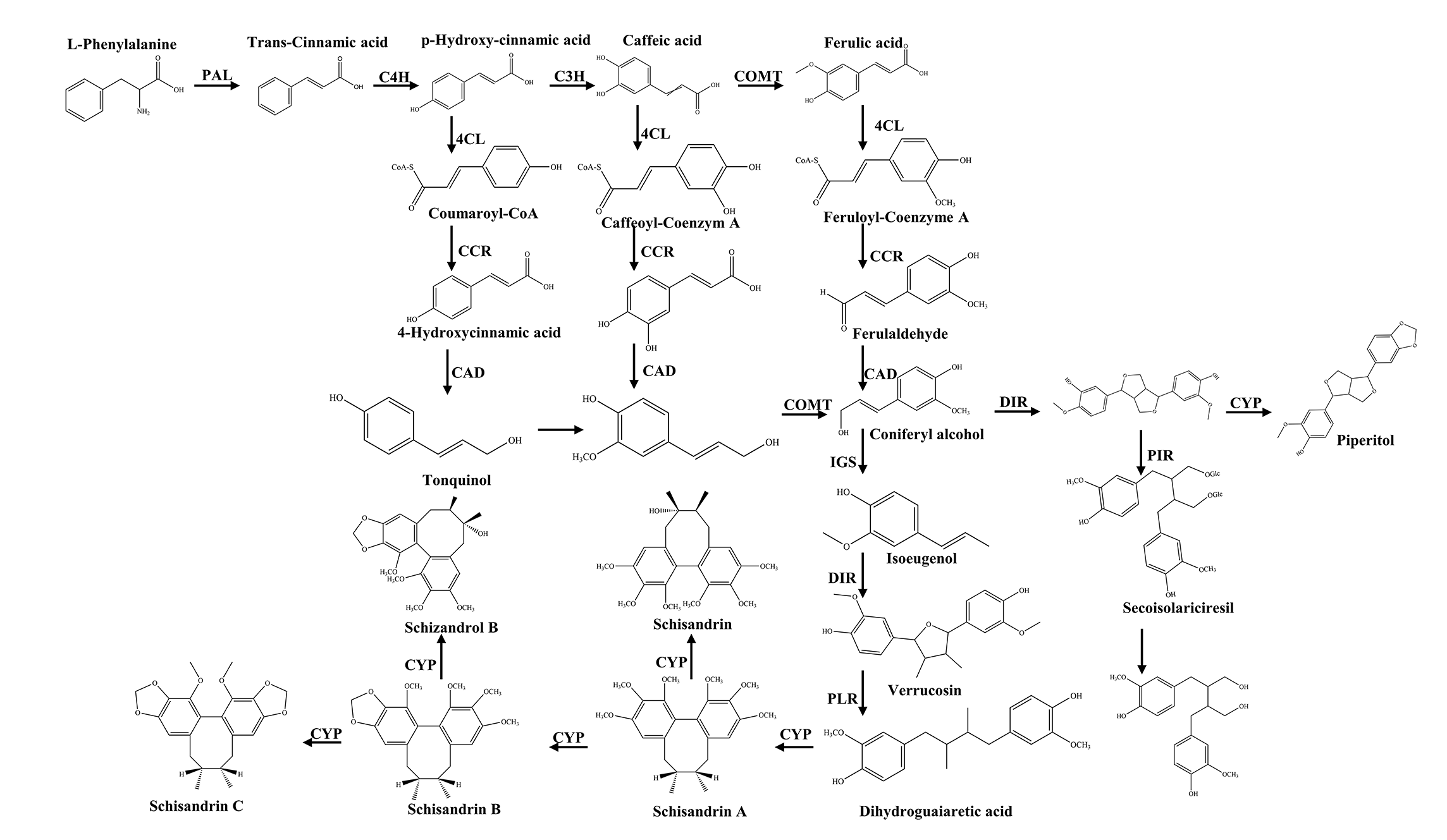


**Supplementary Figure 5.** The biosynthetic pathway of lignans in SCF. (PAL, Phenylalanineammonialyase; C4H, Coumarate-4-hydroxylase; COMT, Caffeic acid-O-methyltransferase; CCR, Cinnamoyl-CoA reductase; CAD, Cinnamyl alcohol dehydrogenase; DIR, Diri-gentprotein; CYP450, Cytochromes P450).


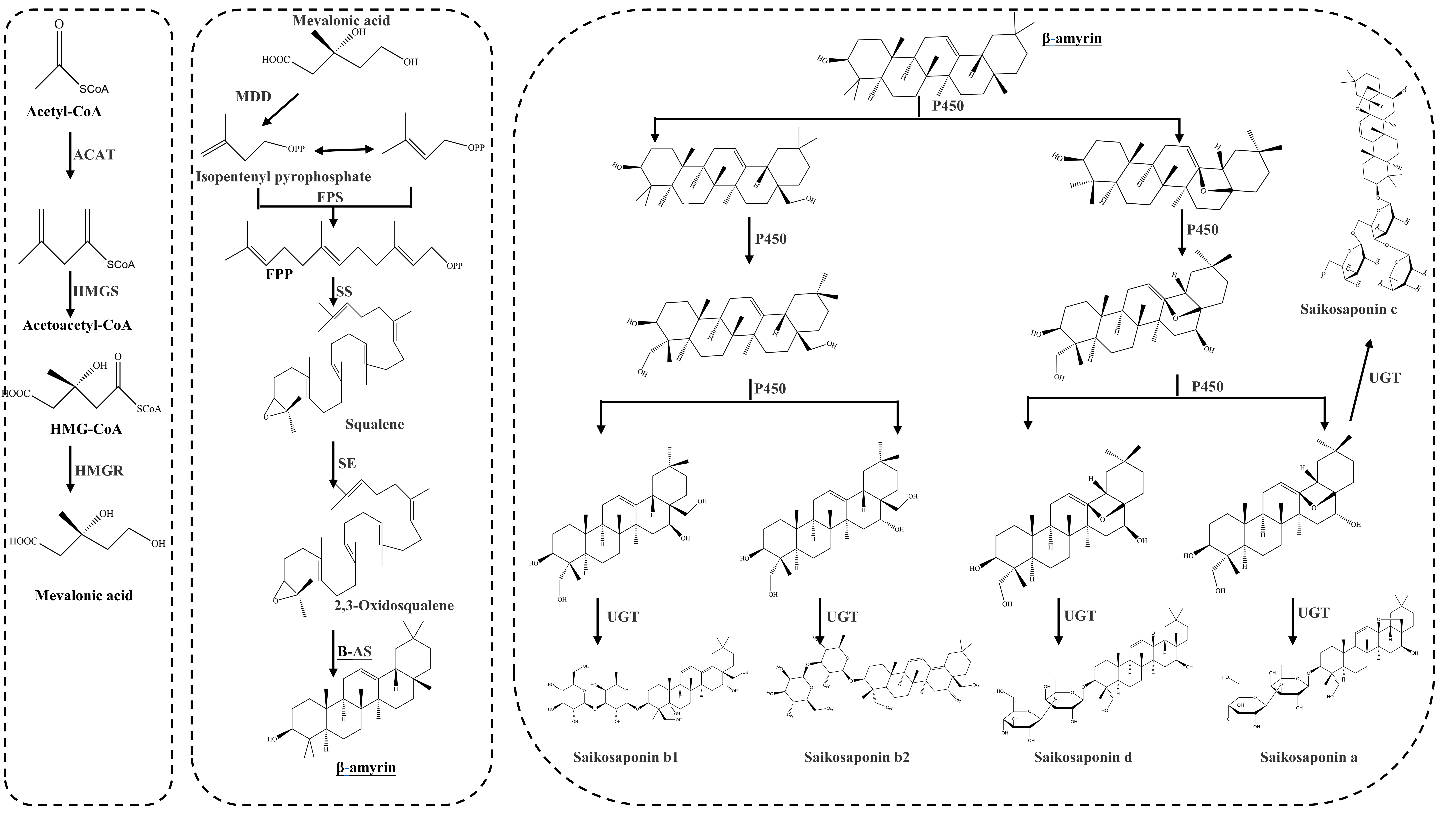


**Supplementary Figure 6.** The biosynthetic pathway of saikosaponins in BR (Acetyl-CoA, Acetyl coenzyme A; Acetoacetyl-CoA, Acetoacetyl coenzyme A; MDD, Mevalonate diphosphate decarboxylase; FPS, Farnesyl diphosphate synthase; SS, Squalene synthase; SE, Squalene epoxidase; UGT, Uridine diphosphate glycosyltransferase).


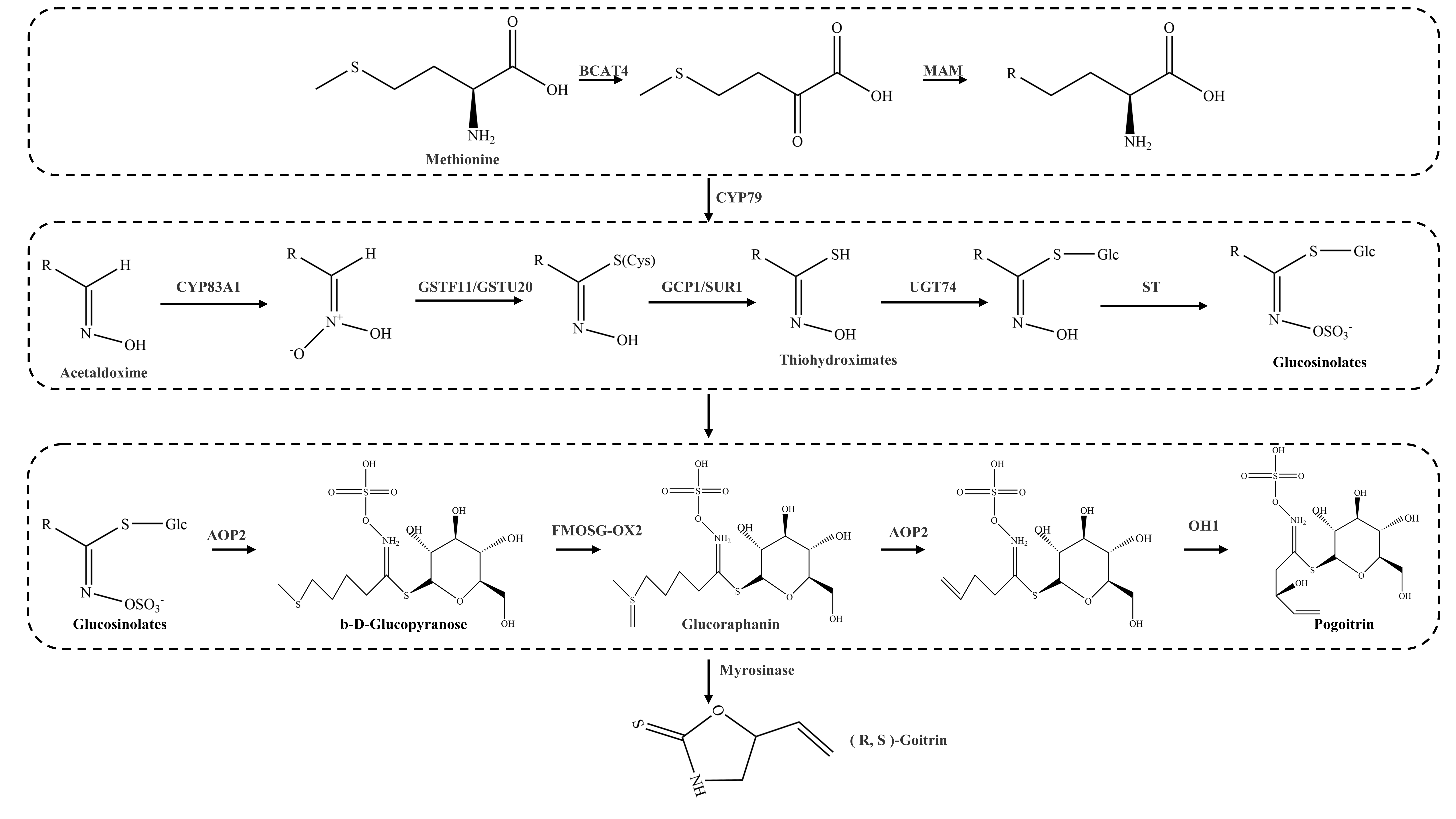


**Supplementary Figure 7.** The biosynthetic pathway of (R, S)-goitrin in IR (BCAT, Phenylalanine ammonia-lyase).


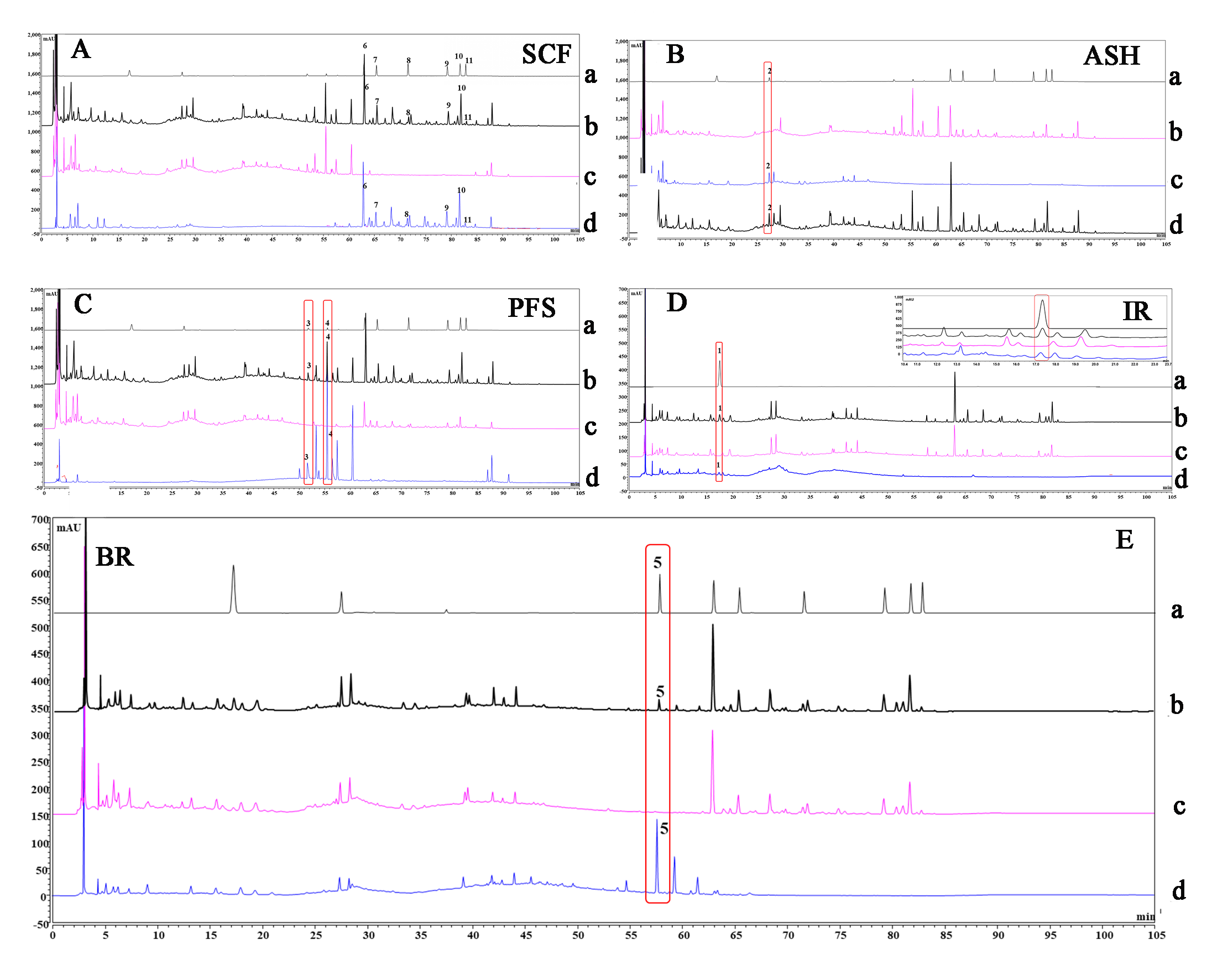


**Supplementary Figure 8.** Specificity research of quantitative analysis **(A, B and C: 200 nm; D and E: 250 nm)**. Note: a - mixed standard solution; b - hugan tablets; c - corresponding herb-negative; d - single herb.


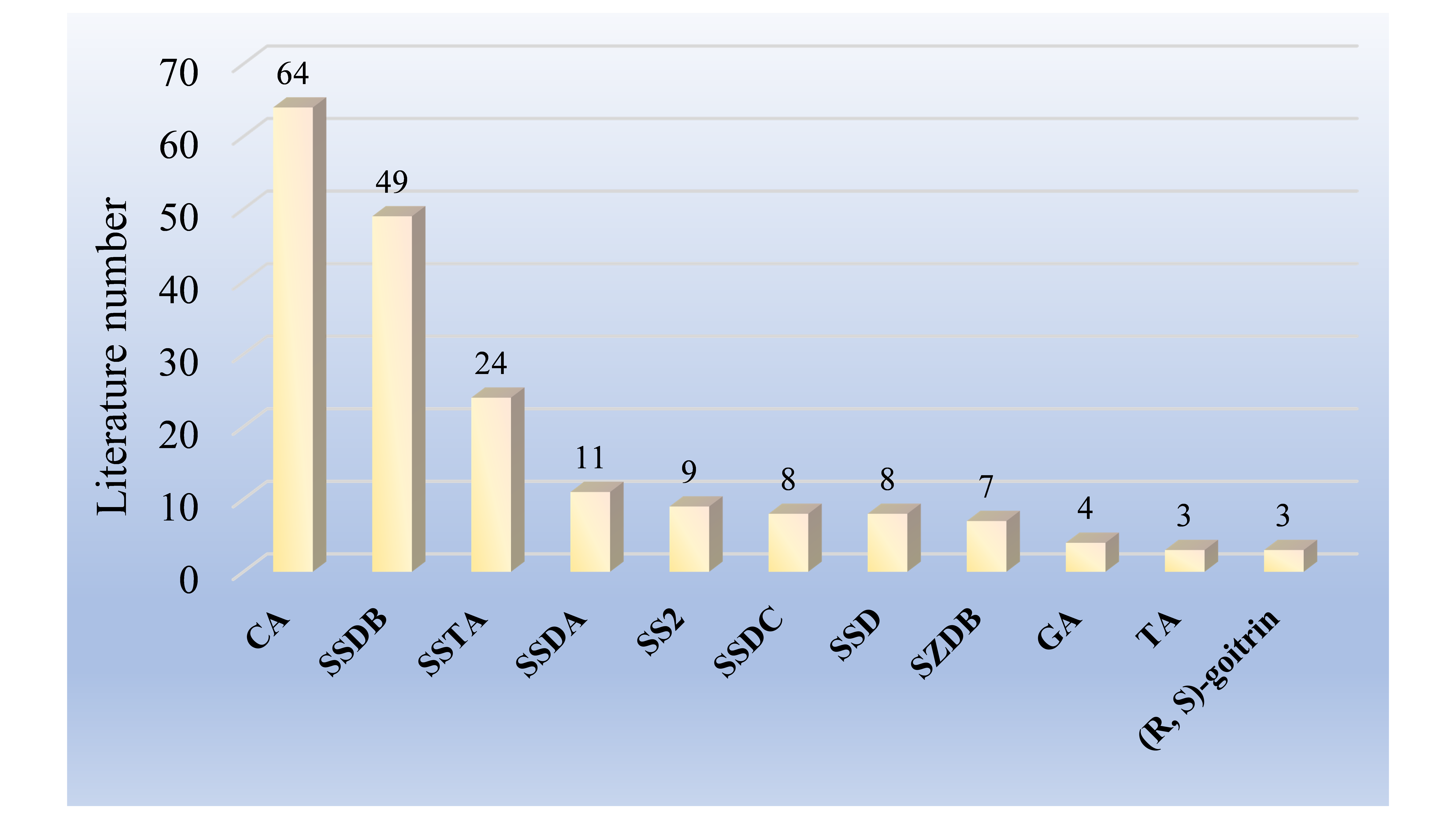


**Supplementary Figure 9.** The number of papers published on the treatment of various liver diseases with 11 plant metabolites in the CNKI, PubMed and Web of Science database up to 2022.

**Supplementary Tables**

**Supplementary Table S1.** Preparation of mixed reference solutions for characteristic chromatogram analysis.

| Reference substance | Concentration (μg·mL^-1^) |  |
| --- | --- | --- |
| (R, S)-Goitrin | 19.41 | Mixed standard solution 1 |
| Guanosine | 14.11 |
| Adenosine | 19.41 |
| Vitexin | 13.53 |
| Chlorogenic acid | 9.41 |
| Rutin | 21.18 |
| Hyperoside | 12.94 |
| Scoparone | 18.82 |
| Quercetin | 15.88 |
| Saikosaponin b2 | 13.53 |
| Schisandrin | 23.53 |
| Schizandrol B | 16.47 |
| Schisantherin A | 12.35 |
| Schisanheno | 24.71 |
| Schisandrin A | 16.47 |
| Schisandrin B | 18.82 |
| Schisandrin C | 26.47 |
| Taurodeoxycholic acid | 40.00 | Mixed standard solution 2 |
| Glycohyodeoxycholic acid | 50.00 |
| Glycochenodeoxycholic acid | 60.00 |

**Supplementary Table S2.** Preparation of mixed reference solution for quantitative analysis.

| Reference substance | Concentration (μg·mL^-1^) |
| --- | --- |
| (R, S)-Goitrin | 0.258 |
| Chlorogenic acid | 0.210 |
| Saikosaponin b2 | 0.153 |
| Taurodeoxycholic acid | 0.600 |
| Glycohyodeoxycholic acid | 2.960 |
| Schisandrin | 1.780 |
| Schizandrol B | 0.330 |
| Schisantherin A | 0.095 |
| Schisandrin A | 0.774 |
| Schisandrin B | 0.290 |
| Schisandrin C | 0.165 |

**Supplementary Table S3.** Active fraction library of Hugan tablet for network pharmacology.

| NO | Molecular formula | Active constituents | Existence form |
| --- | --- | --- | --- |
| 1 | C_5_H_7_NOS | Epigoitrin | + |
| 2 | C_16_H_18_O_9_ | Chlorogenic acid | + |
| 3 | C_7_H_12_O_6_ | Quinic acid | - |
| 4 | C_7_H_10_O_5_ | Shikimic acid | - |
| 5 | C_10_H_12_O_7_S | 4-sulfo-dihydroferulic acid | - |
| 6 | C_10_H_12_O_7_S | 3-sulfo-dihydroisoferulic acid | - |
| 7 | C_10_H_10_O_7_S | 4-sulfo-ferulic acid | - |
| 8 | C_10_H_10_O_7_S | 3-sulfo-isoferulic acid | - |
| 9 | C_9_H_8_O_6_S | 3-sulfo-cinnamic acid | - |
| 10 | C_9_H_10_O_7_S | 4-sulfo-dihydrocaffeic acid | - |
| 11 | C_42_H_68_O_13_ | Saikosaponin b2 | + |
| 12 | C_30_H_48_O_4_ | Saikogenin D | - |
| 13 | C_24_H_32_O_7_ | Schisandrin | + |
| 14 | C_22_H_26_O_7_ | Schisandrin-CH_4_-CH_2_ | - |
| 15 | C_23_H_28_O_7_ | Schizandrol B | + |
| 16 | C_23_H_28_O_8_ | Schizandrol B+OH（1） | - |
| 17 | C_23_H_28_O_8_ | Schizandrol B+OH（2） | - |
| 18 | C_22_H_26_O_7_ | Schizandrol B-CH_2_ | - |
| 19 | C_24_H_32_O_6_ | Schisandrin A | + |
| 20 | C_22_H_26_O_7_ | Schisandrin A*-*O-CH_4_-CH_2_（1） | - |
| 21 | C_22_H_26_O_7_ | Schisandrin A*-*O-CH_4_-CH_2_（2） | - |
| 22 | C_23_H_28_O_8_ | Schisandrin A-C_2_H_5_+COOH | - |
| 23 | C_23_H_26_O_7_ | Schisandrin A-OCH_2_-4H+2O（1） | - |
| 24 | C_23_H_26_O_7_ | Schisandrin A-OCH_2_-4H+2O（2） | - |
| 25 | C_23_H_28_O_6_ | Schisandrin B | + |
| 26 | C_22_H_24_O_6_ | Schisandrin C | + |
| 27 | C_30_H_32_O_9_ | Schisantherin A | + |
| 28 | C_26_H_45_NO_6_S | Taurodeoxycholic acid | + |
| 29 | C_26_H_43_NO_5_ | Glycohyodeoxycholic acid | + |

Note: “+” represents prototype components; “-” represents metabolites.

**Supplementary Table S4.** Compounds identified in Hugan tablets by UPLC-Q-Exactive-Orbitrap/MS.

| No. | Identification | RT  (min) | Formula | Adducts | Observed  (m/z) | MS/MS(*m/z*) | Herb Source |
| --- | --- | --- | --- | --- | --- | --- | --- |
| 1 | Arginine | 0.86 | C_6_H_14_N_4_O_2_ | [M+H]^+^ | 175.1190 | 175.1189[M+H]^+^,  158.0924[M+H-NH_3_]^+^,  112.0869[M+H-HCOOH-NH_3_]^+^,  157.1083[M+H-OH]^+^ | ASH, BR, MB, IR |
| 2 | Proline | 0.92 | C_5_H_9_NO_2_ | [M+H]^+^ | 116.0706 | 116.0706[M+H]^+^,  70.0652[M+H-HCOOH]^+^ | ASH, BR, MB, IR |
| 3 | Valine^#^ | 1.15 | C_5_H_11_NO_2_ | [M+H]^+^ | 118.0863 | 118.0862[M+H]^+^,  72.0808[M+H-HCOOH]^+^ | ASH, MB, IR |
| 4 | Valine^#^ | 1.37 | C_5_H_11_NO_2_ | [M+H]^+^ | 118.0863 | 118.0866[M+H]^+^,  72.0815[M+H-HCOOH]^+^ | ASH, MB, IR |
| 5 | Adenine | 1.37 | C_5_H_5_N_5_ | [M+H]^+^ | 136.0618 | 136.0618[M+H]^+^,  119.0352[M+H-NH]^+^ | ASH, IR, BR, MB |
| 6 | Tyrosine | 1.49 | C_9_H_11_NO_3_ | [M+H]^+^ | 182.0812 | 182.0809[M+H]^+^,  136.0756[M+H-HCOOH]^+^ | ASH, MB, IR |
| 7 | p-coumaric acid | 1.95 | C_9_H_8_O_3_ | [M+H]^+^ | 165.0546 | 165.0544[M+H]^+^,  119.0494[M+H-BROOH]^+^,  123.0443, 95.0496 | ASH, BR, MB, IR |
| 8 | [Isoleucine](javascript:;) | 2.16 | C_6_H_13_NO_2_ | [M+H]^+^ | 132.1019 | 132.1020[M+H]^+^,  86.0964[M+H-HCOOH]^+^ | ASH, BR, MB, IR |
| 9 | [adenosine](javascript:;) | 2.53 | C_10_H_13_N_5_O_4_ | [M+H]^+^ | 268.1040 | 268.1033[M+H]^+^,  136.0618[M+H-BRO]^+^,  119.0355[M+H-C_5_H_8_O_4_-NH_3_]^+^ | ASH, IR, BR, MB |
| 10 | Guanosine* | 2.81 | C_10_H_13_N_5_O_5_ | [M+H]^+^ | 284.0992 | 284.0974[M+H]^+^，  152.0567 [M+H-C_5_H_8_O_4_]^+^ | ASH, IR, BR, MB |
| 11 | Phenylalanine | 3.97 | C_9_H_11_NO_2_ | [M+H]^+^ | 166.0863 | 166.0861[M+H]^+^,  120.0808[M+H-BROOH]^+^,  103.0546 | ASH, MB |
| 12 | 5-hydroxy-2-indolone | 5.42 | C_8_H_7_NO_2_ | [M+H]^+^ | 150.055 | 150.0555[M+H]^+^,  133.0285[M+H-OH]^+^,  132.0448[M+H-H_2_O]^+^ | IR |
| 13 | (*R*, *S*)-goitrin* | 5.62 | C_5_H_7_NOS | [M+H]^+^ | 130.0321 | 130.0323[M+H]^+^,  70.0652[M+H-COS]^+^,84.0444 | IR |

**Supplementary Table S4** (Continued)

| 14 | 3-hydroxymethyl-2-furfural | 5.69 | C_6_H_6_O_3_ | [M+H]^+^ | 127.0390 | 127.0390[M+H]^+^,  109.0285[M+H-H_2_O]^+^ | ASH, IR |
| --- | --- | --- | --- | --- | --- | --- | --- |
| 15 | 1- caffeyl quinic acid | 5.71 | C_16_H_18_O_9_ | [M+H]^+^ | 355.1024 | 355.1027[M+H]^+^,  337.0919[M+H-H_2_O]^+^,  163.0390[M+H-C_7_H_12_O_6_]^+^,  135.1044[M+H-C_7_H_12_O_6_-CO]^+^ | ASH, BR |
| 16 | 4-formyl indole^#^ | 7.09 | C_9_H_7_NO | [M+H]^+^ | 146.0600 | 146.0601[M+H]^+^,  118.0651[M+H-CO]^+^,  117.0575[M+H-BRO]^+^ | ASH, MB |
| 17 | Neochlorogenic acid | 7.16 | C_16_H_18_O_9_ | [M+H]^+^ | 355.1024 | 355.1027[M+H]^+^,  337.0919[M+H-H_2_O]^+^,  163.0390[M+H-C_7_H_12_O_6_]^+^,  135.1044[M+H-C_7_H_12_O_6_-CO]^+^ | ASH, BR |
| 18 | 4-formyl indole^#^ | 7.67 | C_9_H_7_NO | [M+H]^+^ | 146.0600 | 146.0601[M+H]^+^,  118.0653[M+H-CO]^+^, | ASH, BR, IR |
| 19 | Scoparone ^#^ | 7.96 | C_11_H_10_O_4_ | [M+H]^+^ | 207.0652 | 207.0650[M+H]^+^,  179.0699[M+H-CO]^+^，  164.0706[M+H-BR_3_-CO]^+^，  163.0391[M+H-CO_2_]^+^, | ASH, BR |
| 20 | Chlorogenic acid* | 10.15 | C_16_H_18_O_9_ | [M+H]^+^  [M-H]^-^ | 355.1024 (positive)  353.0867 (negative) | postitve：355.1027[M+H]^+^,  337.0919[M+H-H_2_O]^+^,  163.0390[M+H-C_7_H_12_O_6_]^+^,  135.1044[M+H-C_7_H_12_O_6_-CO]^+^  negative：353.0881[M-H]^-^，  191.0562[M-H-C_9_H_6_O_3_]^-^,  179.0354[M-H-C_7_H_10_O_5_]^-^，  173.0458[M-H-C_9_H_6_O_3_-H_2_O]^-^，  127.0402[M-H-C_9_H_6_O_3_-2H_2_O-CO] - | BR, ASH |
| 21 | 7, 8-dihydroxycoumarin | 10.46 | C_9_H_6_O_4_ | [M+H]^+^ | 179.0339 | 179.0339[M+H]^+^,  135.0441[M+H-CO_2_]^+^ | ASH, BR |
| 22 | Scopoletin^#^ | 10.56 | C_10_H_8_O_4_ | [M+H]^+^ | 193.0495 | 193.0495[M+H]^+^，  147.0444[M+H-BR_2_O_2_]^+^，  119.0490[M+H-C_2_H_2_O_3_]^+^ | ASH, BR, MB |

**Supplementary Table S4** (Continued)

| 23 | Cryptochlorogenic acid | 10.88 | C_16_H_18_O_9_ | [M+H]^+^ | 355.1024 | 355.1021[M+H]^+^,  377.0840[M+H-H_2_O]^+^,  163.0390[M+H-C_7_H_12_O_6_]^+^,  135.0443[M+H-C_7_H_12_O_6_-CO]^+^ | ASH, BR |
| --- | --- | --- | --- | --- | --- | --- | --- |
| 24 | 6, 7-dihydroxycoumarin | 8.38 | C_9_H_6_O_4_ | [M+H]^+^ | 179.0339 | 179.0339[M+H]^+^,  135.0445[M+H-CO_2_]^+^ | ASH, BR, MB |
| 25 | Scopoletin^#^ | 12.56 | C_10_H_8_O_4_ | [M+H]^+^ | 193.0495 | 193.0495[M+H]^+^，  119.0491[M+H-H_2_O-CO-CO]^+^,  147.0440[M+H-BR_2_O_2_]^+^， | ASH, BR |
| 26 | p-hydroxyacetophenone | 12.89 | C_8_H_8_O_2_ | [M+H]^+^ | 137.0597 | 137.0598[M+H]^+^,  119.0499[M+H-H_2_O]^+^,  93.0700[M+H-BR_3_BRO]^+^ | ASH, BR |
| 27 | Saffloryellow-A | 13.68 | C_27_H_30_O_15_ | [M+H]^+^ | 595.1657 | 595.1653[M+H]^+^,  577.1541[M+H-H_2_O]^+^,  475.1230[M+H-C_8_H_8_O]^+^ | ASH, MB |
| 28 | 7-Methoxycoumarin^#^ | 14.07 | C_10_H_8_O_3_ | [M+H]^+^ | 177.0546 | 177.0546[M+H]^+^,  163.0389[M+H-BR_2_]^+^，  135.0443[M+H-BR_2_-CO]^+^ | ASH, BR |
| 29 | Quercetin -3- O-rutin glycoside / Quercetin - 3-O-acacia glycoside | 14.63 | C_27_H_30_O_16_ | [M+H]^+^ | 611.1607 | 611.1622[M+H]^+^,  449.1078[M+H-C_6_H_10_O_5_]^+^,  303.0497[M+H-C_6_H_10_O_5_-C_6_H_10_O_4_]^+^ | ASH |
| 30 | Quercetin -3- O-rutin glycoside / Quercetin - 3-O-acacia glycoside | 14.92 | C_27_H_30_O_16_ | [M+H]^+^ | 611.1607 | 611.1622[M+H]^+^,  449.1078[M+H-C_6_H_10_O_5_]^+^,  303.0497[M+H-C_6_H_10_O_5_-C_6_H_10_O_4_]^+^ | ASH |
| 31 | Scopoletin^#^ | 15.29 | C_10_H_8_O_4_ | [M+H]^+^ | 193.0495 | 193.0496[M+H]^+^，  151.0754[M+H-BR_2_-CO]^+^,  147.0437[M+H-H_2_O-CO]^+^，  119.0857[M+H-H_2_O-CO-CO]^+^ | BR, ASH, MB |
| 32 | 7-methoxy coumarin^#^ | 15.44 | C_10_H_8_O_3_ | [M+H]^+^ | 177.0546 | 177.0545[M+H]^＋^,  163.0387[M+H-BR_2_]^＋^，  135.0439[M+H-BR_2_-CO]^＋^ | ASH, BR |

**Supplementary Table S4** (Continued)

| 33 | Quercetin - 3-O-glucoside/ isoquercitrin | 15.74 | C_21_H_20_O_12_ | [M+H]^+^ | 465.1027 | 465.1052[M+H]^＋^,  303.0495[M+H-C_6_H_10_O_5_]^+^,  165.0177[M+H-C_6_H_10_O_5_-C_7_H_6_O_3_]^+^,  153.0180[M+H-C_14_H_16_O_8_]^+^,  137.0233[M+H-C_14_H_16_O_9_]^+^ | ASH |
| --- | --- | --- | --- | --- | --- | --- | --- |
| 34 | Vitexin* | 16.14 | C_21_H_20_O_10_ | [M+H]^+^ | 433.1129 | 433.1125[M+H]^+^,  415.1027[M+H-H_2_O]^+^,  397.0915[M+H-2H_2_O]^+^,  379.0800[M+H-3H_2_O]^+^,  271.0603[M+H-H_2_O-C_6_H_9_O_4_]^+^ | MB, ASH |
| 35 | Rutin* | 16.23 | C_27_H_30_O_16_ | [M+H]^+^ | 611.1607 | 611.1559[M+H]^+^，  465.1032[M+H-C_6_H_10_O_4_]^+^,  303.0498[M+H-C_6_H_10_O_5_-C_6_H_10_O_4_]^+^,  285.0387[M+H-C_6_H_10_O_5_-C_6_H_10_O_4_-H_2_O]^+^,  229.0499[M+H-C_6_H_10_O_5_-C_6_H_10_O_4_-H_2_O-2CO]^+^，  257.0445[M+H-C_6_H_10_O_5_-C_6_H_10_O_4_-H_2_O-CO]^+^,  165.0185[M+H-C_6_H_10_O_5_-C_6_H_10_O_4_-C_7_H_6_O_3_]^+^,  153.0186[M+H-C_6_H_10_O_5_-C_6_H_10_O_4_-C_8_H_6_O_3_]^+^,  137.0236[M+H-C_6_H_10_O_5_-C_6_H_10_O_4_-C_8_H_6_O_4_]^+^ | BR, MB, ASH |
| 36 | Isovitexin | 16.33 | C_21_H_20_O_10_ | [M+H]^+^ | 433.1129 | 433.1126[M+H]^+^,  415.1021[M+H-H_2_O]^+^,  397.0914[M+H-2H_2_O]^+^,  379.0807[M+H-3H_2_O]^+^,  271.0598[M+H-H_2_O-C_6_H_9_O_4_]^+^ | MB, ASH, IR |
| 37 | Hyperin* | 16.37 | C_21_H_20_O_12_ | [M+H]^+^ | 465.1028 | 487.0855[M+Na]^+^,  465.0990[M+H]^+^,  303.0495[M+H-C_6_H_10_O_5_]^+^,  257.0439[M+H-C_6_H_10_O_5_-C_6_H_10_O_4_-H_2_O-CO]^+^，  165.0177[M+H-C_6_H_10_O_5_-C_7_H_6_O_3_]^+^ | BR, ASH |

**Supplementary Table S4** (Continued)

| 38 | Isoquercitrin / Quercetin - 3-O-glucoside | 16.62 | C_21_H_20_O_12_ | [M+H]^+^ | 465.1028 | 465.1052[M+H]^+^,  303.0498[M+H-C_6_H_10_O_5_]^+^，  165.0184[M+H-C_6_H_10_O_5_-C_7_H_6_O_3_]^+^,  153.0182[M+H-C_14_H_16_O_8_] ^+^,  137.0233[M+H-C_14_H_16_O_9_] ^+^ | BR, ASH, MB |
| --- | --- | --- | --- | --- | --- | --- | --- |
| 39 | Dicaffeoylquinic acid | 17.39 | C_25_H_24_O_12_ | [M+H]^+^  [M-H]^-^ | 517.1341 (positive)  515.1184 (negative) | 正：517.1656[M+H]^+^,  449.6078[M+H-H_2_O]^+^,  163.0390[M+H-C_9_H_9_O_3_-C_7_H_12_O_6_]^+^  负：515.1194, 353.0885, 191.0563 | ASH, BR |
| 40 | Narcissin^#^ | 17.63 | C_28_H_32_O_16_ | [M+H]^+^ | 625.1763 | 647.1589[M+Na]^+^,  625.1758[M+H]^+^，  479.1184[M+H-C_6_H_10_O_4_]^+^，  317.0647[M+H-C_6_H_10_O_5_-C_6_H_10_O_4_]^+^ | ASH |
| 41 | Morin | 17.75 | C_15_H_10_O_7_ | [M+H]^+^ | 303.0499 | 303.0495[M+H]^+^,  285.0385[M+H-H_2_O] ^+^,  257.0431[M+H-H_2_O-CO]^+^,  229.0483[M+H-H_2_O-2CO]^+^,  165.0178[M+H-C_7_H_6_O_3_]^+^,  153.0184[M+H-C_8_H_6_O_3_]^+^,  137.0235[M+H-C_8_H_6_O_4_]^+^ | ASH, BR |
| 42 | Dicaffeoylquinic acid | 17.84 | C_25_H_24_O_12_ | [M+H]^+^  [M-H]^-^ | 517.1341 (positive)  515.1184 (negative) | 正：517.1656[M+H]^+^,  449.6078[M+H-H_2_O]^+^,  163.0390[M+H-C_9_H_9_O_3_-C_7_H_12_O_6_]^+^  负：515.1207, 447.0941, 353.0883, 191.0564 | ASH, BR |
| 43 | Narcissin^#^ | 17.86 | C_28_H_32_O_16_ | [M+H]^+^ | 625.1763 | 647.1591[M+Na]^+^,  625.1687[M+H]^+^，  479.1187[M+H-C_6_H_10_O_4_]^+^，  317.0652[M+H-C_6_H_10_O_5_-C_6_H_10_O_4_]^+^ | BR, ASH |
| 44 | Capillarisin | 18.19 | C_16_H_12_O_7_ | [M+H]^+^ | 317.0656 | 317.0650[M+H]^+^，  273.0394[M+H-C_6_H_4_O]^+^,  302.0408[M+H-BR_3_]^+^，  285.0415[M+H-BR_3_OH]^+^ | ASH |

**Supplementary Table S4** (Continued)

| 45 | Isorhamnetin | 18.40 | C_16_H_12_O_7_ | [M+H]^+^ | 317.0656 | 317.0650[M+H]^+^，  302.0415[M+H-BR_3_] ^+^，  285.0391[M+H-BR_3_-OH] ^+^，  274.0469[M+H-BR_3_-CO] ^+^，  257.0429[M+H-BR_3_-OH-CO] ^+^，  229.0490[M+H-BR_3_-OH-2CO] ^+^，  217.0520[M+H-BR_3_-OH-2CO-C] ^+^，  153.0184[M+H-C_9_H_8_O_3_] ^+^ | ASH, MB, BR |
| --- | --- | --- | --- | --- | --- | --- | --- |
| 46 | Dicaffeoylquinic acid | 18.76 | C_25_H_24_O_12_ | [M+H]^+^  [M-H]^-^ | 517.1341 (positive)  515.1184 (negative) | Positive: 517.1656[M+H]^+^,  449.6078[M+H-H2O]^+^,  163.0390[M+H-C9H9O3-C7H12O6]^+^  Negative: 515.1193353.0883, 191.0563 | ASH, BR |
| 47 | Scoparone* | 18.92 | C_11_H_10_O_4_ | [M+H]^+^ | 207.0652 | 207.0652[M+H]^+^,  192.0417[M+H-BR_3_]^+^,  179.0704[M+H-CO]^+^,  163.0386[M+H-CO_2_]^+^,  151.0755[M+H-2CO]^+^,  136.0521[M+H-BR_3_-2CO]^+^ | BR, ASH |
| 48 | Quercetin^#^ | 20.35 | C_15_H_10_O_7_ | [M+H]^+^ | 303.0499 | 303.0492[M+H]^+^，  257.0435[M+H-H_2_O-CO]^+^,  229.0491[M+H-H_2_O-2CO]^+^,  165.0174[M+H-C_7_H_6_O_3_]^+^,  153.0181[M+H-C_8_H_6_O_3_]^+^,  137.0227[M+H-C_8_H_6_O_4_]^+^ | BR, ASH |
| 49 | Genkwanin | 22.24 | C_16_H_12_O_5_ | [M+H]^+^ | 285.0758 | 285.0754[M+H]^+^，  270.0523[M+H-BR_3_]^＋^ | ASH |

**Supplementary Table S4** (Continued)

| 50 | Quercetin* | 22.78 | C_15_H_10_O_7_ | [M+H]^+^ | 303.0499 | 303.0495[M+H]^+^，  257.0441[M+H-H_2_O-CO]^+^,  229.0502[M+H-H_2_O-2CO]^+^,  165.0184[M+H-C_7_H_6_O_3_]^+^,  153.0182[M+H-C_8_H_6_O_3_]^+^,  137.0236[M+H-C_8_H_6_O_4_]^+^ | BR, ASH |
| --- | --- | --- | --- | --- | --- | --- | --- |
| 51 | Carvacrol^#^ | 27.42 | C_11_H_16_ | [M+H]^+^ | 149.1325 | 149.1325[M+H]^+^,  121.1012[M+H-2BR_2_] ^+^,  107.0855[M+H-3BR_2_] ^+^ | ASH, BR |
| 52 | Tauro-6-oxo-lithoBRolic acid | 28.06 | C_26_H_43_NO_6_S | [M+H]^+^  [M-H]^-^ | 498.2884  496.2709 | 498.2895[M+H]^+^  496.2740[M-H]^-^ | PFS |
| 53 | Limettin | 28.54 | C_11_H_10_O_4_ | [M+H]^+^ | 207.0652 | 207.0650[M+H]^+^,  192.0415[M+H-BR_3_]^+^,  164.0467[M+H-BR_3_-CO]^+^,  147.0442[M+H-C_3_H_4_O_2_]^+^,  119.0491 M+H-C_3_H_4_O_3_]^+^, | BR |
| 54 | Carvacrol^#^ | 28.81 | C_11_H_16_ | [M+H]^+^ | 149.1325 | 149.1324[M+H]^+^,  121.1011[M+H-2BR_2_] ^+^,  107.0854[M+H-3BR_2_] ^+^ | ASH, BR |
| 55 | Saikosaponin F | 29.48 | C_48_H_80_O_17_ | [M+H]^+^ | 929.5468 | 951.5287[M+Na]^+^,  929.5486[M+H]^+^,  603.4231[M+H-C_6_H_10_O_5_-C_6_H_10_O_4_-H_2_O]^+^, 585.4096[M+H-C_6_H_10_O_5_-C_6_H_10_O_4_-2H_2_O]^+^, 423.3621[M+H-2C_6_H_10_O_5_-C_6_H_10_O_4_-2H_2_O]^+^ | BR |
| 56 | Henridilactone B | 30.06 | C_29_H_34_O_11_ | [M+H]^+^ | 559.2174 | 559.2173[M+H]^+^,  541.2068[M+H-H_2_O]^+^，  497.2154[M+H-H2O-CO_2_]^+^, 453.2269 | SCF |
| 57 | Henridilactone A | 30.51 | C_29_H_34_O_10_ | [M+H]^+^ | 543.2225 | 565.2045[M+Na]^+^,  543.2227[M+H]^+^, 499.2323[M+H-CO_2_]^+^ | SCF |
| 58 | Schindilactone A | 30.70 | C_29_H_34_O_10_ | [M+H]^+^ | 543.2225 | 543.2238[M+H]^+^,  525.2120[M+H-H_2_O]^+^,  483.2011, 465.1891, 455.2041 | SCF |

**Supplementary Table S4** (Continued)

| 59 | Saikosaponin B2* | 31.31 | C_42_H_68_O_13_ | [M+H]^+^  [M-H]^-^ | 781.4733 (positive)  779.4576 (negative) | 正：803.4542[M+Na]^+^,  781.4720[M+H]^+^，  619.4263[M+H-C_6_H_10_O_5_]^+^, 763.4612, 745.4513,  601.4066[M+H-C_6_H_10_O_5_-H_2_O]^+^，  455.3521[M+H-C_6_H_10_O_5_-C_6_H_10_O_4_-H_2_O]^+^,  437.3412[M+H-C_6_H_10_O_5_-C_6_H_10_O_4_-H_2_O]^+^，  419.3288[M+H-C_6_H_10_O_5_-C_6_H_10_O_4_-3H_2_O]^+^  负：779.4631, 617.4083 | BR |
| --- | --- | --- | --- | --- | --- | --- | --- |
| 60 | Lancifodilactone D | 31.56 | C_29_H_34_O_9_ | [M+H]^+^ | 527.2276 | 549.2094[M+Na]^+^, 527.2277[M+H]^+^,  509.2171[M+H-H_2_O]^+^,  467.2064, 449.1952 | SCF |
| 61 | Schisandrin* | 32.26 | C_24_H_32_O_7_ | [M+H]^+^ | 433.2221 | 433.2216[M+H]^+^,  415.2114[M+H-H_2_O]^+^，  384.1930[M+H-H_2_O-OBR_3_]^+^,  369.1690[M+H-H_2_O-OBR_3_-BR_3_]^+^ | SCF |
| 62 | Gomisin R^#^ | 33.05 | C_22_H_24_O_7_ | [M+H]^+^ | 401.1595 | 401.1576[M+H]^+^,  383.1480[M+H-H_2_O]^+^,  352.1303, 341.1013[M+H-H_2_O-C_3_H_6_]^+^, 371.1469 | SCF |
| 63 | Gomisin D | 33.14 | C_28_H_34_O_10_ | [M+H]^+^ | 531.2225 | 531.2222[M+H]^+^,  485.2169[M+H-BR_2_O_2_]^+^,  401.1591[M+H-C_6_H_10_O_3_]^+^,  383.1484[M+H-C_6_H_10_O_3_-H_2_O]^+^,  341.1009[M+H-C_6_H_10_O_3_-H_2_O-C_3_H_6_]^+^ | SCF |
| 64 | Gomisin J | 33.32 | C_22_H_28_O_6_ | [M+H]^+^ | 389.1959 | 389.1954[M+H]^+^，  357.1695[M+H-BR_3_OH]^+^,  287.0910[M+H-BR_3_OH-C_5_H_10_]^+^,  227.0701[M+H-2BR_3_OH-C_5_H_10_-CO]^+^ | SCF |
| 65 | Schizandrol B* | 33.53 | C_23_H_28_O_7_ | [M+H]^+^ | 417.1908 | 417.1900[M+H]^+^,  399.1799[M+H-H_2_O]^+^,  368.1616[M+H-H_2_O-OBR_3_]^+^,  353.1385[M+H-H_2_O-OBR_3_-BR_3_]^+^ | SCF |
| 66 | Gomisin R^#^ | 33.75 | C_22_H_24_O_7_ | [M+H]^+^ | 401.1595 | 401.1590[M+H]^+^,  383.1486[M+H-H_2_O]^+^,  352.1301, 341.1015[M+H-H_2_O-C_3_H_6_]^+^, 371.1460 | SCF |

**Supplementary Table S4** (Continued)

| 67 | Linolenic acid^#^ | 34.08 | C_18_H_30_O_2_ | [M+H]^+^ | 279.2319 | 279.2317[M+H]^+^,  195.1391[M+H-C_6_H_12_]^+^,  133.1009[M+H-C_10_H_18_O_2_]^+^  119.0857[M+H-C_9_H_20_O_2_]^+^  105.0703[M+H-C_10_H_22_O_2_]^+^  95.0855[M+H-C_11_H_20_O_2_]^+^ | SCF |
| --- | --- | --- | --- | --- | --- | --- | --- |
| 68 | Linolenic acid^#^ | 34.35 | C_18_H_30_O_2_ | [M+H]^+^ | 279.2319 | 279.2312[M+H]^+^,  195.1373[M+H-C_6_H_12_]^+^,  133.1009[M+H-C_10_H_18_O_2_]^+^  119.0857[M+H-C_9_H_20_O_2_]^+^  105.0703[M+H-C_10_H_22_O_2_]^+^  95.0855[M+H-C_11_H_20_O_2_]^+^ | SCF |
| 69 | Saikosaponin F | 34.6 | C_28_H_36_O_8_ | [M+H]^+^ | 501.2483 | 501.2462[M+H]^+^,  483.2386[M+H-H_2_O]^+^,  401.1964[M+H-C_4_H_7_COOH]^+^,  370.1785[M+H-C_4_H_7_COOH-OBR_3_]^+^, 355.1945[M+H-C_4_H_7_COOH-OBR_3_-BR_3_]^+^, 386.1733[M+H-C_4_H_7_COOH-BR_3_]^+^, 523.2302[M+Na]^+^ | SCF |
| 70 | [Pregomisin](https://www.chemsrc.com/en/cas/66280-26-0_1367250.html) | 34.67 | C_22_H_30_O_6_ | [M+H]^+^ | 391.2115 | 391.2112[M+H]^+^,  237.1483[M+H-C_8_H_10_O_3_]^+^,  205.1222[M+H-C_8_H_10_O_3_-BR_3_OH]^+^,  167.0703[M+H-C_13_H_20_O_3_]^+^ | SCF |
| 71 | Gomisin O/Epigomisin O | 35.11 | C_23_H_28_O_7_ | [M+H]^+^ | 417.1907 | 417.1855[M+H]^+^,  399.1726[M+H-H_2_O]^+^,  353.1263[M+H-H_2_O-OBR_3_-BR_3_]^+^ | SCF |
| 72 | **Angeloylgomisin H** | 35.29 | C_28_H_36_O_8_ | [M+H]^+^ | 501.2483 | 483.2378[M+H-H_2_O]^+^,  401.1962[M+H-C_4_H_7_COOH]^+^,  370.1772[M+H-C_4_H_7_COOH-OBR_3_]^+^, 355.1543[M+H-C_4_H_7_COOH-OBR_3_-BR_3_]^+^, 386.1733[M+H-C_4_H_7_COOH-BR_3_]^+^ | SCF |
| 73 | Benzoylgomisin H | 35.59 | C_30_H_34_O_8_ | [M+H]^+^ | 523.2326 | 523.2323[M+H]^+^，  505.2221[M+H-H_2_O]^+^,  401.1942[M+H-C_6_H_5_COOH]^+^ | SCF |

**Supplementary Table S4** (Continued)

| 74 | Benzoylgomisin Q | 35.93 | C_31_H_36_O_9_ | [M+H]^+^ | 553.2432 | 553.2453[M+H]^+^,  431.2061[M+H-C_6_H_5_COOH]^+^,  387.1802[M+H-C_6_H_5_COOH-C_2_H_4_O]^+^,  341.1376[M+H-C_6_H_5_COOH-C_2_H_4_O-OBR_3_-BR_3_]^+^,  356.1614[M+H-C_6_H_5_COOH-C_2_H_4_O-OBR_3_]^+^ | SCF |
| --- | --- | --- | --- | --- | --- | --- | --- |
| 75 | Schisantherin A^#^ | 35.99 | C_30_H_32_O_9_ | [M+H]^+^ | 537.2119 | 537.2097[M+H]^+^，  415.1752[M+H-C_6_H_5_COOH]^+^，  371.1487[M+H-C_6_H_5_COOH-C_2_H_4_O]^+^,  340.1301[M+H-C_6_H_5_COOH-C_2_H_4_O-OBR_3_]^+^，  325.1072[M+H-C_6_H_5_COOH-C_2_H_4_O-OBR_3_-BR_3_]^+^ | SCF |
| 76 | Gomisin G^#^ | 35.89 | C_30_H_32_O_9_ | [M+H]^+^ | 537.2119 | 537.2100[M+H]^+^，  415.1746[M+H-C_6_H_5_COOH]^+^，  371.1490[M+H-C_6_H_5_COOH-C_2_H_4_O]^+^，  340.1303[M+H-C_6_H_5_COOH-C_2_H_4_O-OBR_3_]^+^,  325.1065[M+H-C_6_H_5_COOH-C_2_H_4_O-OBR_3_-BR_3_]^+^,  356.1283[M+H-C_6_H_5_COOH-C_2_H_4_O-BR_3_]^+^,  559.1938[M+Na]^+^ | SCF |
| 77 | Gomisin G^#^ | 36.19 | C_30_H_32_O_9_ | [M+H]^+^ | 537.2119 | 537.2100[M+H]^+^,  415.1745[M+H-C_6_H_5_COOH]^+^,  371.1488[M+H-C_6_H_5_COOH-C_2_H_4_O]^+^,  340.1305[M+H-C_6_H_5_COOH-C_2_H_4_O-OBR_3_]^+^,  325.1077[M+H-C_6_H_5_COOH-C_2_H_4_O-OBR_3_-BR_3_]^+^,  356.1283[M+H-C_6_H_5_COOH-C_2_H_4_O-BR_3_]^+^,  559.1938[M+Na]^+^ | SCF |
| 78 | Gomisin G^#^ | 36.29 | C_30_H_32_O_9_ | [M+H]^+^ | 537.2119 | 537.2100[M+H]^+^,  415.1745[M+H-C_6_H_5_COOH]^+^,  371.1499[M+H-C_6_H_5_COOH-C_2_H_4_O]^+^,  340.1302[M+H-C_6_H_5_COOH-C_2_H_4_O-OBR_3_]^+^, 325.1083[M+H-C_6_H_5_COOH-C_2_H_4_O-OBR_3_-BR_3_]^+^,  356.1283[M+H-C_6_H_5_COOH-C_2_H_4_O-BR_3_]^+^,  559.1938[M+Na]^+^ | SCF |

**Supplementary Table S4** (Continued)

| 79 | Gomisin K1 | 36.32 | C_23_H_30_O_6_ | [M+H]^+^ | 403.2115 | 403.2112[M+H]^+^,  333.1333[M+H-C_5_H_10_]^+^,  371.1841[M+H-BR_3_OH]^+^,  302.1145[M+H-OBR_3_-C_5_H_10_]^+^,  287.0920[M+H-OBR_3_-C_5_H_10_-BR_3_]^+^,  227.0700[M+H-OBR_3_-C_5_H_10_-BR_3_-C_2_H_4_O_2_]^+^,  301.1070[M+H-BR_3_OH-C_5_H_10_]^+^, | SCF |
| --- | --- | --- | --- | --- | --- | --- | --- |
| 80 | Gomisin K2 | 36.49 | C_23_H_30_O_6_ | [M+H]^+^ | 403.2115 | 403.2112[M+H]^+^,  371.1847[M+H-BR_3_OH]^+^,  333.1327[M+H-C_5_H_10_]^+^,  340.1664[M+H-BR_3_OH-OBR_3_]^+^,  302.1143[M+H-OBR_3_-C_5_H_10_]^+^,  301.1071[M+H-BR_3_OH-C_5_H_10_]^+^, | SCF |
| 81 | Schisantherin A* | 36.84 | C_30_H_32_O_9_ | [M+H]^+^ | 537.2119 | 537.2108[M+H]^+^,  415.1749[M+H-C_6_H_5_COOH]^+^,  371.1480[M+H-C_6_H_5_COOH-C_2_H_4_O]^+^,  340.1299[M+H-C_6_H_5_COOH-C_2_H_4_O-OBR_3_]^+^,  325.1067[M+H-C_6_H_5_COOH-C_2_H_4_O-OBR_3_-BR_3_]^+^,  356.1246[M+H-C_6_H_5_COOH-C_2_H_4_O-BR_3_]^+^,  559.1938[M+Na]^+^ | SCF |
| 82 | Schisantherin A^#^ | 37.01 | C_30_H_32_O_9_ | [M+H]^+^ | 537.2119 | 537.2097[M+H]^+^,  415.1748[M+H-C_6_H_5_COOH]^+^,  371.1494[M+H-C_6_H_5_COOH-C_2_H_4_O]^+^,  340.1302[M+H-C_6_H_5_COOH-C_2_H_4_O-OBR_3_]^+^,  325.1068[M+H-C_6_H_5_COOH-C_2_H_4_O-OBR_3_-BR_3_]^+^,  356.1253[M+H-C_6_H_5_COOH-C_2_H_4_O-BR_3_]^+^,  559.1938[M+Na]^+^ | SCF |
| 83 | Gomisin M1 / M2  or Gomisin L1 / L2 | 37.09 | C_22_H_26_O_6_ | [M+H]^+^ | 387.1802 | 387.1795[M+H]^+^,  372.1556,  355.1536[M+H-BR_3_OH]^+^,  227.0705[M+H-BR_3_OH-C_5_H_10_-C_2_H_2_O_2_]^+^,  255.0654 | SCF |

**Supplementary Table S4** (Continued)

| 84 | Gomisin M1 / M2  or Gomisin L1 / L2 | 37.25 | C_22_H_26_O_6_ | [M+H]^+^ | 387.1802 | 387.1797[M+H]^+^,372.1559,  355.1539[M+H-BR_3_OH]^+^,  285.0757[M+H-BR_3_OH-C_5_H_10_]^+^,  227.0700[M+H-BR_3_OH-C_5_H_10_-C_2_H_2_O_2_]^+^ | SCF |
| --- | --- | --- | --- | --- | --- | --- | --- |
| 85 | Linolenic acid^#^ | 37.28 | C_18_H_30_O_2_ | [M+H]^+^ | 279.2319 | 279.2315[M+H]^+^,  195.1380[M+H-C_6_H_12_]^+^,  133.1009[M+H-C_10_H_18_O_2_]^+^  119.0857[M+H-C_9_H_20_O_2_]^+^  105.0703[M+H-C_10_H_22_O_2_]^+^  95.0855[M+H-C_11_H_20_O_2_]^+^ | BR, ASH |
| 86 | Schisanhenol* | 37.35 | C_23_H_30_O_6_ | [M+H]^+^ | 403.2115 | 403.2112[M+H]^+^,  388.1881,  371.1841[M+H-BR_3_OH]^+^,  340.1662[M+H-BR_3_OH-OBR_3_]^+^,  302.1140, 301.1071 | SCF |
| 87 | Linolenic acid^#^ | 37.37 | C_18_H_30_O_2_ | [M+H]^+^ | 279.2319 | 279.2318[M+H]^+^,  195.1387[M+H-C_6_H_12_]^+^,  133.1009[M+H-C_10_H_18_O_2_]^+^  119.0857[M+H-C_9_H_20_O_2_]^+^  105.0703[M+H-C_10_H_22_O_2_]^+^  95.0855[M+H-C_11_H_20_O_2_]^+^ | BR, ASH |
| 88 | Gomisin M1, M2  or L1, L2 | 37.51 | C_22_H_26_O_6_ | [M+H]^+^ | 387.1802 | 387.1799[M+H]^+^,  372.1559,355.1537[M+H-BR_3_OH]^+^，  285.0756[M+H-BR_3_OH-C_5_H_10_]^+^,  227.0702[M+H-BR_3_OH-C_5_H_10_-C_2_H_2_O_2_]^+^ | SCF |
| 89 | Tigloylgomisin P / Angeloylgomisin P | 38.04 | C_28_H_34_O_9_ | [M+H]^+^ | 515.2276 | 515.2275[M+H]^+^,  385.1643[M+H-C_4_H_7_COOH-OBR_3_]^+^,  355.1537，340.1129 | SCF |
| 90 | Gomisin M1 / M2  or Gomisin L1 / L2 | 38.07 | C_22_H_26_O_6_ | [M+H]^+^ | 387.1802 | 387.1799[M+H]^+^,  372.1555,  355.1535[M+H-BR_3_OH]^+^，  285.0752[M+H-BR_3_OH-C_5_H_10_]^+^,  227.0703[M+H-BR_3_OH-C_5_H_10_-C_2_H_2_O_2_]^+^ | SCF |

**Supplementary Table S4** (Continued)

| 91 | Gomisin E | 38.15 | C_28_H_34_O_9_ | [M+H]^+^ | 515.2276 | 515.2276[M+H]^+^,  385.1643[M+H-C_6_H_10_O_3_]^+^,  355.1541[M+H-C_6_H_10_O_3_-BR_2_O]^+^, 316.0945[M+H-C_6_H_10_O_3_-C_5_H_9_]^+^,  469.2222[M+H-BR_2_O_2_]^+^ | SCF |
| --- | --- | --- | --- | --- | --- | --- | --- |
| 92 | Gomisin M1 / M2  or Gomisin L1 / L2 | 38.37 | C_22_H_26_O_6_ | [M+H]^+^ | 387.1802 | 387.1795[M+H]^+^,  372.1559,355.1533[M+H-BR_3_OH]^+^，285.0757[M+H-BR_3_OH-C_5_H_10_]^+^,  227.0701[M+H-BR_3_OH-C_5_H_10_-C_2_H_2_O_2_]^+^ | SCF |
| 93 | Schisandrin A* | 39.48 | C_24_H_32_O_6_ | [M+H]^+^ | 417.2272 | 417.2269[M+H]^+^,  370.1785[M+H-C_2_H_7_O]^+^,  402.2036,  386.2089[M+H-OBR_3_]^+^,  316.1300[M+H-C_5_H_10_-OBR_3_]^+^,  301.1069[M+H-C_5_H_10_-OBR_3_-BR_3_]^+^, 285.1118[M+H-C_5_H_10_-2OBR_3_]^+^,  242.0939[M+H-C_5_H_10_-2OBR_3_-C_2_H_3_O]^+^, 227.0700[M+H-C_5_H_10_-2OBR_3_-C_2_H_3_O-BR_3_]^+^, 347.1488[M+H-C_5_H_10_]+ | SCF |
| 94 | γ-schizandrin | 40.28 | C_23_H_28_O_6_ | [M+H]^+^ | 401.1959 | 401.1956[M+H]^+^,  386.1720[M+H-BR_3_]^+^,  371.1848[M+H-BR_2_O]^+^,  331.1178[M+H-C_5_H_10_]^+^,  300.0989[M+H-C_5_H_10_-OBR_3_]^+^,  316.0945[M+H-C_5_H_1_0-BR_3_]^+^,  423.1775[M+Na]^+^ | SCF |
| 95 | Schisandrin B* | 40.54 | C_23_H_28_O_6_ | [M+H]^+^ | 401.1959 | 401.1955[M+H]^+^,  386.1720[M+H-BR_3_]^+^,  371.1857[M+H-BR_2_O]^+^,  331.1168[M+H-C_5_H_10_]^+^,  300.0987[M+H-C_5_H_10_-OBR_3_]^+^,  316.0950[M+H-C_5_H_1_0-BR_3_]^+^,  423.1774[M+Na]^+^ | SCF |

**Supplementary Table S4** (Continued)

| 96 | Schisandrin C* | 41.06 | C_22_H_24_O_6_ | [M+H]^+^ | 385.1646 | 385.1640[M+H]^+^,  355.1538[M+H-BR_2_O]^+^,  315.0841[M+H-C_5_H_10_]^+^,  285.0753[M+H-C_5_H_10_-BR_2_O]^+^,  299.0907,  257.0809[M+H-C_5_H_10_-C_2_H_2_O_2_]^+^,  199.0758[M+H-C_5_H_10_-2C_2_H_2_O_2_]^+^ | SCF |
| --- | --- | --- | --- | --- | --- | --- | --- |
| 97 | Benzoylgomisin O | 41.66 | C_30_H_32_O_8_ | [M+H]^+^ | 521.2170 | 521.2132[M+H]^+^,  399.1809[M+H-C_6_H_5_COOH]^+^,  369.1696, 330.1092, 299.0918 | SCF |
| 98 | Kadsuric acid | 44.40 | C_30_H_46_O_4_ | [M+H]^+^ | 471.3469 | 471.3470[M+H]^+^,  453.3353[M+H-H_2_O]^+^,  145.1016[M+H-C_19_H_34_O_4_]^+^ | SCF |
| 99 | Nigranoic acid | 44.77 | C_30_H_46_O_4_ | [M+H]^+^ | 471.3469 | 471.3470[M+H]^+^,  453.3347[M+H-H_2_O]^+^,  145.1014[M+H-C_19_H_34_O_4_]^+^ | SCF |
| 100 | Schisanlactone D | 45.32 | C_30_H_44_O_3_ | [M+H]^+^ | 453.3363 | 453.3375[M+H]^+^,  435.3257[M+H-H_2_O]^+^，  145.1014[M+H-C_19_H_31_O_3_]^+^,  203.1794[M+H-C_15_H_21_O_3_]^+^,  313.2519[M+H-C_8_H_12_O_2_]^+^ | SCF |
| 101 | Saponarin | 12.75 | C_27_H_30_O_15_ | [M-H]^-^ | 593.1501 | 593.1517[M-H]^-^, 473.1093, 311.0563 | IR |
| 102 | Clemastanin B | 13.28 | C_32_H_44_O_16_ | [M-H]^-^ | 683.2546 | 683.2592[M-H]^-^,  521.2026[M-H-C_6_H_10_O_5_]^-^,  329.1395[M-H-C_13_H_22_O_11_]^-^，  359.1505[M-H-C_12_H_20_O_10_]^-^ | IR |
| 103 | Isovitexin 6'-O-glucoside | 14.44 | C_27_H_30_O_15_ | [M-H]^-^ | 593.1501 | 593.1516[M-H]^-^, 473.1063, 341.0666, 311.0563 | IR |
| 104 | Isoplantagenin-6 '-o-glucopyranoside | 14.97 | C_28_H_32_O_16_ | [M-H]^-^ | 623.1607 | 623.1634[M-H]^-^, 371.0785, 341.0672 | IR |
| 105 | IndigoticosideA | 15.55 | C_26_H_34_O_11_ | [M-H]^-^ | 521.2017 | 521.2027[M-H]^-^,  329.1396[M-H-C_7_H_12_O_6_]^-^,  359.1504[M-H-C_6_H_10_O_5_]^-^ | IR, ASH, MB |

**Supplementary Table S4** (Continued)

| 106 | (+)-Isolariciresinol 9'-O-glucoside | 16.29 | C_26_H_34_O_11_ | [M-H]^-^ | 521.2017 | 521.2021[M-H]^-^,  329.1396[M-H-C_7_H_12_O_6_]^-^,  359.1500[M-H-C_6_H_10_O_5_^]-^ | IR, ASH, MB |
| --- | --- | --- | --- | --- | --- | --- | --- |
| 107 | Isoscoparin | 16.91 | C_22_H_22_O_11_ | [M-H]^-^ | 461.1078 | 461.1092[M-H]^-^, 371.0779, 341.0668 | IR |
| 108 | kaempferol | 22.81 | C_15_H_10_O_6_ | [M-H]^-^ | 285.0394 | 285.0404[M-H]^-^, 151.0041 | ASH, MB |
| 109 | Taurohyocholic acid | 25.79 | C_26_H_45_NO_7_S | [M-H]^-^ | 514. 2833 | 514.2842[M-H]^-^ | PFS |
| 110 | Taurohyodeoxycholic acid * | 27.40 | C_26_H_45_NO_6_S | [M-H]^-^ | 498.2884 | 498.2892[M-H]^-^ | PFS |
| 111 | Glycocholic Acid | 28.75 | C_26_H_43_NO_6_ | [M-H]^-^ | 464. 3007 | 464.3019[M-H]^-^,  420.3134[M-H-CO_2_]^-^, 400.2879, 74.0247 | PFS |
| 112 | Glycohyodeoxycholic acid* | 29.80 | C_26_H_43_NO_5_ | [M-H]^-^ | 448.3057 | 448.3071[M-H]^-^,  386.3052 [M-H-H_2_O-CO_2_]^-^,74.0247 | PFS |
| 113 | Taurochenodeoxycholic Acid | 29.83 | C_26_H_45_NO_6_S | [M-H]^-^ | 498.2884 | 498.2894[M-H]^-^ | PFS |
| 114 | Glycocholic Acid | 30.08 | C_26_H_43_NO_6_ | [M-H]^-^ | 464.3007 | 464.3021[M-H]^-^,  402.3015[M-H-H_2_O-CO_2_]^-^, 400.2863, 74.0247 | PFS |
| 115 | **Saikosaponin B3** / **Saikosaponin B4** | 30.16 | C_43_H_72_O_14_ | [M-H]^-^ | 811.4838 | 811.4849[M-H]^-^，  649.4305[M-H-C_6_H_10_O_5_]^-^,  857.5031[M+HCOO]^-^ | BR |
| 116 | **Saikosaponin S** | 30.82 | C_59_H_74_O_10_ | [M-H]^-^ | 941.5198 | 987.5157[M+HCOO]^-^，  941.5157[M-H]^-^，  617.4104[M-H-2(C_6_H_10_O_5_-H_2_O)]^-^ | BR |
| 117 | Hyocholic acid | 31.16 | C_24_H_40_O_5_ | [M-H]^-^ | 407.2792 | 407.2806[M-H]^-^,  389.2695[M-H-H_2_O]^-^, | PFS |
| 118 | **Saikosaponin** a | 31.20 | C_42_H_68_O_13_ | [M-H]^-^ | 779.4576 | 779.4631[M-H]^-^,  617.4083[M-H-C_6_H_10_O_5_]^-^ | BR |
| 119 | Hyodeoxycholic acid | 31.50 | C_24_H_40_O_4_ | [M-H]^-^ | 391.2843 | 783.5818[2M-H]^-^,  391.2859[M-H]^-^，  373.2744[M-H-H_2_O]^-^ | PFS |

**Supplementary Table S4** (Continued)

| 120 | Cholic acid | 31.88 | C_24_H_40_O_5_ | [M-H]^-^ | 407.2792 | 407.2806[M-H]^-^,  343.2643[M-H-CO-2H_2_O]^-^, 289.2166 | PFS |
| --- | --- | --- | --- | --- | --- | --- | --- |
| 121 | **Saikosaponin** g | 31.89 | C_42_H_68_O_13_ | [M-H]^-^ | 779.4576 | 779.4630[M-H]^-^,  736.6020,  617.4070[M-H-C_6_H_10_O_5_]^-^,  471.3473[M-H-Fuc-Glc]^-^ | BR |
| 122 | **Saikosaponin** b1 | 32.02 | C_42_H_68_O_13_ | [M-H]^-^ | 779.4576 | 779.4596[M-H]^-^,  617.4071[M-H-C_6_H_10_O_5_]^-^ | BR |
| 123 | Glycochenodeoxycholic acid* | 32.17 | C_26_H_43_NO_5_ | [M-H]^-^ | 448.3057 | 448.3071[M-H]^-^,  386.3074[M-H-H_2_O-CO_2_]^-^,  74.0247 | PFS |
| 124 | Chenodeoxycholic acid | 32.36 | C_24_H_40_O_4_ | [M-H]^-^ | 391.2843 | 391.2859[M-H]^-^,  373.2755[M-H-H_2_O]^-^,  783.5774[2M-H]^-^ | SCF |
| 125 | 6''-O-Acetylsaikosaponin A | 33.08 | C_44_H_70_O_14_ | [M-H]^-^ | 821.4682 | 821.4703[M-H]^-^,  779.4589[M-H-C_2_H_2_O]^-^,  617.4066[M-H-C_2_H_2_O-Glc]^-^ | BR |
| 126 | 6''-O-Acetylsaikosaponin A | 33.80 | C_44_H_70_O_14_ | [M-H]^-^ | 821.4682 | 821.4706[M-H]^-^,  779.4564[M-H-C_2_H_2_O]^-^,  617.4071[M-H-C_2_H_2_O-Glc]^-^ | BR |
| 127 | Deoxycholic acid | 35.03 | C_24_H_40_O_4_ | [M-H]^-^ | 391.2843 | 391.2858[M-H]^-^,  373.2758[M-H-H_2_O]^-^,  783.5778[2M-H]^-^ | PFS |
| 128 | Glycolithocholic acid | 35.64 | C_26_H_43_O_4_N | [M-H]^-^ | 432.3108 | 432.3124[M-H]^-^,  388.3244[M-H-CO_2_]^-^, | PFS |

Note：BR, Bupleuri Radix; YH, Artemisiae Scopariae Herba; IR, Isatidis Radix; WWZ, Schisandrae chinensis Fructus; MB, mung bean; PFS, Pulvis Fellis Suis.

a*: Confirmation in comparison with reference standards. Isomeric forms were distinguished by a#.

**Supplementary Table S5.** The study on the precision, repeatability and stability of characteristic chromatogram.

| NO | Relative retention time RSD (%) | | | Relative peak area RSD (%) | | |
| --- | --- | --- | --- | --- | --- | --- |
|  | Precision | Repeatability | Stability | Precision | Repeatability | Stability |
| 1 | 0.06% | 0.08% | 0.07% | 2.3% | 0.6% | 4.3% |
| 2 | 0.03% | 0.07% | 0.04% | 2.6% | 0.6% | 2.5% |
| 3 | 0.05% | 0.07% | 0.06% | 2.7% | 1.3% | 2.0% |
| 4 | 0.02% | 0.07% | 0.03% | 3.0% | 0.9% | 3.0% |
| 5 | 0.02% | 0.05% | 0.02% | 2.7% | 3.7% | 4.3% |
| 6 | 0.00% | 0.00% | 0.00% | 2.9% | 0.7% | 3.0% |
| 7 | 0.00% | 0.00% | 0.00% | 0.0% | 0.0% | 0.0% |
| 8 | 0.00% | 0.00% | 0.00% | 2.5% | 0.3% | 2.5% |
| 9 | 0.00% | 0.00% | 0.01% | 2.4% | 0.3% | 2.5% |
| 10 | 0.08% | 0.12% | 0.09% | 1.1% | 0.9% | 0.5% |
| 11 | 0.02% | 0.04% | 0.02% | 1.2% | 0.8% | 1.0% |
| 12 | 0.05% | 0.02% | 0.07% | 3.5% | 4.9% | 3.0% |
| 13 | 0.06% | 0.03% | 0.08% | 1.3% | 1.0% | 1.1% |
| 14 | 0.01% | 0.01% | 0.01% | 2.2% | 1.2% | 0.9% |
| 15 | 0.01% | 0.01% | 0.02% | 1.6% | 1.3% | 1.7% |
| 16 | 0.01% | 0.01% | 0.01% | 0.2% | 0.5% | 0.1% |
| 17 | 0.00% | 0.01% | 0.01% | 0.6% | 1.1% | 0.7% |
| 18 | 0.01% | 0.01% | 0.01% | 3.3% | 0.7% | 3.0% |
| 19 | 0.01% | 0.01% | 0.01% | 0.3% | 0.5% | 0.2% |
| 20 | 0.00% | 0.00% | 0.00% | 0.0% | 0.0% | 0.0% |
| 21 | 0.00% | 0.00% | 0.00% | 0.7% | 0.9% | 0.3% |
| 22 | 0.00% | 0.00% | 0.00% | 0.3% | 0.3% | 0.3% |
| 23 | 0.00% | 0.00% | 0.00% | 0.4% | 0.3% | 0.2% |
| 24 | 0.00% | 0.00% | 0.00% | 1.0% | 0.5% | 1.0% |
| 25 | 0.00% | 0.00% | 0.00% | 0.1% | 0.2% | 0.1% |
| 26 | 0.00% | 0.00% | 0.00% | 0.2% | 0.3% | 0.2% |
| 27 | 0.00% | 0.00% | 0.00% | 0.1% | 0.3% | 0.1% |
| 28 | 0.00% | 0.00% | 0.00% | 0.3% | 0.5% | 0.4% |
| 29 | 0.01% | 0.01% | 0.01% | 3.9% | 2.6% | 3.7% |
| 30 | 0.00% | 0.00% | 0.00% | 0.8% | 1.2% | 0.7% |
| 31 | 0.00% | 0.00% | 0.00% | 0.2% | 0.2% | 0.2% |
| 32 | 0.00% | 0.01% | 0.00% | 0.1% | 0.6% | 0.1% |
| 33 | 0.00% | 0.01% | 0.00% | 0.1% | 0.5% | 0.1% |
| 34 | 0.00% | 0.00% | 0.00% | 0.3% | 1.9% | 0.3% |
| 35 | 0.00% | 0.01% | 0.01% | 2.7% | 3.7% | 0.8% |

**Supplementary Table S6.** The study on the durability of the characteristic chromatogram.

| NO | Relative retention time RSD (%) | | |
| --- | --- | --- | --- |
|  | Flow rate | Column temperature | Columns |
| 1 | 6.5% | 2.4% | 12% |
| 2 | 2.7% | 2.3% | 6.7% |
| 3 | 3.2% | 2.3% | 6.0% |
| 4 | 2.1% | 1.4% | 6.3% |
| 5 | 1.5% | 1.1% | 6.5% |
| 6 | 0.1% | 0.1% | 0.3% |
| 7 | 0.0% | 0.0% | 0.0% |
| 8 | 0.1% | 0.1% | 0.1% |
| 9 | 0.1% | 0.1% | 0.1% |
| 10 | 7.0% | 1.4% | 4.6% |
| 11 | 1.0% | 0.5% | 2.6% |
| 12 | 0.2% | 0.1% | 1.7% |
| 13 | 0.3% | 0.3% | 0.3% |
| 14 | 0.2% | 0.1% | 0.3% |
| 15 | 0.2% | 0.1% | 0.3% |
| 16 | 0.3% | 0.1% | 0.2% |
| 17 | 0.3% | 0.1% | 0.3% |
| 18 | 0.3% | 0.1% | 0.2% |
| 19 | 0.3% | 0.1% | 0.2% |
| 20 | 0.0% | 0.0% | 0.1% |
| 21 | 0.1% | 0.1% | 0.2% |
| 22 | 0.1% | 0.2% | 0.2% |
| 23 | 0.1% | 0.1% | 0.0% |
| 24 | 0.2% | 0.1% | 0.3% |
| 25 | 0.2% | 0.1% | 0.2% |
| 26 | 0.2% | 0.2% | 0.3% |
| 27 | 0.2% | 0.2% | 0.3% |
| 28 | 0.2% | 0.2% | 0.3% |
| 29 | 0.2% | 0.2% | 0.3% |
| 30 | 0.3% | 0.3% | 0.3% |
| 31 | 0.2% | 0.2% | 0.3% |
| 32 | 0.3% | 0.3% | 0.4% |
| 33 | 0.3% | 0.3% | 0.4% |
| 34 | 0.3% | 0.3% | 0.4% |
| 35 | 0.4% | 0.3% | 0.5% |

**Supplementary Table S7.** Identification results of characteristic peaks of Hugan tablets.

| NO | Compounds | *t*_R_  (min) | Molecular  formula | Observed  (*m*/*z*) | MS/MS(*m*/*z*) | Herb Source |
| --- | --- | --- | --- | --- | --- | --- |
| 1 | (*R*, *S*)-goitrin | 17.32 | C_5_H_7_NOS | 130.0321 | 130.0323, 70.0658, 84.0450 | IR |
| 4 | Quercetin -3- O-rutin glycoside / Quercetin - 3-O-acacia glycoside | 35.03 | C_27_H_30_O_16_ | 611.1607 | 611.1622, 449.1078, 303.0497 | ASH |
| 5 | Quercetin -3- O-rutin glycoside / Quercetin - 3-O-acacia glycoside | 36.11 | C_27_H_30_O_16_ | 611.1607 | 661.1609, 449.1078, 303.0497 | ASH |
| 6 | Saikosaponin b3/Saikosaponin b4 | 55.13 | C_43_H_72_O_14_ | 811.4849 | 811.4849, 649.4305, 857.4901 | BR |
| 7 | Saikosaponin b2 | 57.72 | C_42_H_68_O_13_ | 781.4733 | 803.4542, 781.4720, 763.4612, 601.4095,455.3514, 437.3409, 437.3415, 419.3309 | BR |
| 9 | Saikosaponin b1 | 61,14 | C_42_H_68_O_13_ | 781.4733 | 781.4720, 763.4621, 745.4485, 455.3524,437.3409, 437.3417, 419.3314 | BR |
| 12 | Taurohyocholic acid | 51.01 | C_26_H_45_NO_7_S | 514.2833 | 514.2836, 487.3520, 407.6370, 319.3964 | PFS |
| 13 | Taurohyodeoxycholic acid | 52.83 | C_26_H_45_NO_6_S | 498.2884 | 498.2887, 432.3156, 472.5658 | PFS |
| 14 | Tauro-6-oxo-lithoBRolic acid/isomer | 53.85 | C_26_H_43_NO_6_S | 496.2709 | 496.2735 | PFS |
| 15 | Glycocholic acid or isomeride | 54.43 | C_26_H_43_NO_6_ | 464.3007 | 464.3001, 420.3112, 400.2853, 389.1618 | PFS |
| 16 | Glycohyodeoxycholic acid | 55.91 | C_26_H_43_NO_5_ | 448.3057 | 448.3070, 386.3069, 74.0234 | PFS |
| 17 | Cholyglycine | 56.91 | C_26_H_43_NO_6_ | 464. 3007 | 464.3004, 446.2905, 402.2991 | PFS |
| 18 | Hyocholic acid | 57.80 | C_24_H_40_O_5_ | 407.2792 | 407,2876, 389.2701, 371.2583 | PFS |
| 19 | Glycochenodeoxycholic acid | 60.42 | C_26_H_43_NO_5_ | 448.3057 | 448.3062, 386.3064, 74.0234 | PFS |
| 20 | Schisandrin | 62.68 | C_24_H_32_O_7_ | 433.2221 | 433.2282, 415.2115, 384.1929, 369.1697 | SCF |
| 21 | Gomisin D | 63.49 | C_28_H_34_O_10_ | 531.2225 | 531.2222, 485.2170, 401.1595, 383.1489, 341.1017 | SCF |
| 22 | Gomisin J | 64.08 | C_22_H_28_O_6_ | 389.1959 | 389.1956, 370.3105, 357.1698, 287.0912, 227.0703 | SCF |
| 23 | Schizandrol B | 64.81 | C_23_H_28_O_7_ | 417.1908 | 417.1899, 399.1803, 375.2803, 368.1610, 384.1569 | SCF |

**Supplementary Table S7** (Continued)

| 24 | Saikosaponin H | 66.09 | C_28_H_36_O_8_ | 501.2483 | 501.2462, 483.2386, 401.1964, 370.1785, 355.1945, 386.1733, 523.2302 | SCF |
| --- | --- | --- | --- | --- | --- | --- |
| 25 | Benzoylgomisin H | 67.36 | C_28_H_36_O_8_ | 501.2483 | 483.2378, 401.1962, 370.1772, 355.1543, 386.1733, | SCF |
| 26 | Schisantherin A | 68.50 | C_30_H_32_O_9_ | 537.2119 | 537.2688, 415.1748, 371.1491, 340.1307, 325.1068, 356.1253, 559.1938 | SCF |
| 27 | Schisantherin A or isomeride | 68.61 | C_30_H_32_O_9_ | 537.2119 | 537.2688, 415.1748, 371.1491, 340.1307, 325.1068, 356.1253, 559.1938 | SCF |
| 31 | Schisandrin A | 75.23 | C_24_H_32_O_6_ | 417.2272 | 417.2271, 370.1765, 402.2041, 386.2083, 316.1305, 301.1070, 285.1118, 242.0942, 227.0700, 347.1489 | SCF |
| 32 | γ-schizandrin | 76.50 | C_23_H_28_O_6_ | 401.1959 | 401.1958, 386.1720, 371.1848, 331.1164, 300.0991, 316.0945, 423.1775 | SCF |
| 33 | Schisandrin B | 76.97 | C_23_H_28_O_6_ | 401.1959 | 401.1958, 386.1729, 371.1857, 331.1171, 300.0989, 316.0950, 423.1774 | SCF |
| 34 | Schisandrin C | 77.74 | C_22_H_24_O_6_ | 385.1646 | 385.1639, 355.1532, 315.0855, 285.0755, 299.0907, 257.0807, 199.0752 | SCF |

Note：BR, Bupleuri Radix; YH, Artemisiae Scopariae Herba; IR, Isatidis Radix; WWZ, Schisandrae chinensis Fructus; MB, mung bean; PFS, Pulvis Fellis Suis.

**Supplementary Table S8.** Contents of 11 specific components in 15 batches of Hugan tablets (*n*=2).

| NO | Content (%) | | | | | | | | | | |
| --- | --- | --- | --- | --- | --- | --- | --- | --- | --- | --- | --- |
|  | (R, S)-goitrin | CA | TA | GA | Sb2 | Schizandrin | Schizandrol B | Schisandrin A | Schisandrin B | Schisandrin C | Schisantherin A |
| 1 | 0.024% | 0.042% | 0.189% | 1.314% | 0.024% | 0.222% | 0.072% | 0.060% | 0.113% | 0.013% | 0.019% |
| 2 | 0.022% | 0.041% | 0.179% | 1.264% | 0.021% | 0.209% | 0.069% | 0.059% | 0.110% | 0.012% | 0.019% |
| 3 | 0.024% | 0.039% | 0.175% | 1.238% | 0.022% | 0.213% | 0.072% | 0.059% | 0.111% | 0.012% | 0.019% |
| 4 | 0.025% | 0.043% | 0.181% | 1.294% | 0.023% | 0.204% | 0.069% | 0.057% | 0.106% | 0.013% | 0.018% |
| 5 | 0.025% | 0.039% | 0.176% | 1.258% | 0.023% | 0.199% | 0.065% | 0.055% | 0.100% | 0.011% | 0.018% |
| 6 | 0.025% | 0.035% | 0.170% | 1.179% | 0.022% | 0.176% | 0.057% | 0.049% | 0.089% | 0.010% | 0.016% |
| 7 | 0.027% | 0.041% | 0.184% | 1.287% | 0.023% | 0.196% | 0.064% | 0.055% | 0.101% | 0.012% | 0.017% |
| 8 | 0.024% | 0.040% | 0.160% | 1.280% | 0.023% | 0.183% | 0.058% | 0.050% | 0.092% | 0.010% | 0.017% |
| 9 | 0.024% | 0.036% | 0.149% | 1.192% | 0.022% | 0.172% | 0.054% | 0.048% | 0.088% | 0.010% | 0.016% |
| 10 | 0.022% | 0.032% | 0.137% | 1.097% | 0.020% | 0.155% | 0.051% | 0.042% | 0.078% | 0.009% | 0.014% |
| 11 | 0.022% | 0.038% | 0.151% | 1.172% | 0.022% | 0.188% | 0.059% | 0.055% | 0.098% | 0.011% | 0.017% |
| 12 | 0.021% | 0.038% | 0.142% | 1.172% | 0.022% | 0.181% | 0.055% | 0.054% | 0.094% | 0.010% | 0.017% |
| 13 | 0.021% | 0.038% | 0.175% | 1.306% | 0.023% | 0.189% | 0.058% | 0.057% | 0.099% | 0.010% | 0.018% |
| 14 | 0.022% | 0.037% | 0.163% | 1.280% | 0.023% | 0.176% | 0.054% | 0.057% | 0.097% | 0.010% | 0.018% |
| 15 | 0.021% | 0.037% | 0.165% | 1.270% | 0.022% | 0.176% | 0.055% | 0.052% | 0.091% | 0.010% | 0.017% |
| Average | 0.023% | 0.038% | 0.166% | 1.240% | 0.022% | 0.189% | 0.061% | 0.054% | 0.098% | 0.011% | 0.017% |
| RSD | 8.0% | 7.5% | 9.5% | 5.1% | 4.4% | 9.5% | 11.6% | 9.1% | 9.8% | 11.3% | 7.9% |

**Supplementary Table S9.** Prototypical components and metabolites of Hugan tablet in rat serum.

| NO | Identification | Formula | *t*_R_ (min) | *m/z* | MS/MS information |
| --- | --- | --- | --- | --- | --- |
| (*R*, *S*)-goitrin | | | | | |
| M1-0 | (*R*, *S*)-goitrin | C_5_H_7_NOS | 5.42 | 130.0321 | 130.0322[M+H]^+^, 84.0811,  70.0655[M+H-COS]^+^ |
| Chlorogenic acid | | | | | |
| M2-0 | Chlorogenic acid | C_16_H_18_O_9_ | 10.23 | 353.0867 | 353.0886[M-H]^-^, 191.0564， |
| M2-1 | Quinic acid | C_7_H_12_O_6_ | 0.91 | 191.055 | 191.0560[M-H]^-^  111.0088[M-H-H_2_O-CO_2_-H_2_O]^-^,  173.0092[M-H-H_2_O]^-^,  85.0295 [M-H-H_2_O-CO_2_-C_2_H_4_O]^‑^ |
| M2-2 | Shikimic acid | C_7_H_10_O_5_ | 1.67 | 173.0444 | 173.0448[M-H]^-^  111.0089[M-H-H_2_O-CO_2_-H_2_O]^-^,  85.0295 [M-H-H_2_O-CO_2_-C_2_H_4_O]^-^ |
| M2-3 | 4-sulfo-dihydroferulic acid/3-sulfo-dihydroisoferulic acid | C_10_H_12_O_7_S | 9.99 | 275.0220 | 275.0233[M-H]^-^，  195.0663[M-H-SO_3_]^-^, 135.0455 |
| M2-4 | 4-sulfo-ferulic acid/3-sulfo-isoferulic acid | C_10_H_10_O_7_S | 10.88 | 273.0063 | 273.0066[M-H]^-^，  193.0509[M-H-SO_3_]^-^，  178.0272[M-H-SO_3_-CH_3_]^-^,  134.0373 [M-H-SO_3_-CH_3_-CO_2_]^-^ |
| M2-5 | 3-sulfo-cinnamic acid | C_9_H_8_O_6_S | 11.47 | 242.9958 | 242.9965[M-H]^-^,  163.0404[M-H-SO_3_]^-^，  119.0503 [M-H-SO_3_-CO_2_]^-^ |
| M2-6 | 4-sulfo-dihydrocaffeic acid | C_9_H_10_O_7_S | 13.92 | 261.0063 | 261.0077[M-H]^-^,  181.0509[M-H-SO_3_]^-^ |

**Supplementary Table S9** (Continued)

| Saikosaponin b2 | | | | | |
| --- | --- | --- | --- | --- | --- |
| M3-0 | Saikosaponin b2 | C_42_H_68_O_13_ | 31.26 | 779.4576 | 825.4631[M+HCOO]^-^,  779.4626[M-H]^-^，  617.4078 [M-H-Glc]^-^ |
| M3-1 | Saikogenin D | C_30_H_48_O_4_ | 36.56 | 473.3625 | 455.2965[M-H_2_O+H]^+^ ,  437.2863[M-2H_2_O+H]^+^ |
| Schisandrin | | | | | |
| M4-0 | Schisandrin | C_24_H_32_O_7_ | 32.22 | 433.2221 | 433.2254[M+H]^+^,  415.2131[M+H-H_2_O]^+^,  384.1935[M+H-H_2_O-OCH_3_]^+^,  369.1712[M+H-H_2_O-OCH_3_-CH_3_]^+^ |
| M4-1 | Schisandrin-CH_4_-CH_2_ | C_22_H_26_O_7_ | 20.14 | 403.1751 | 403.1744[M+H]^+^,  371.1500[M+H-H_2_O-CH_2_]^+^, 353.1399 |
| Schizandrol B | | | | | |
| M5-0 | schizandrol B | C_23_H_28_O_7_ | 33.52 | 417.1908 | 417.1901[M+H]^+^,  399.1819[M+H-H_2_O]^+^,  369.1712[M+H-H_2_O-CH_2_O]^+^, |
| M5-1 | schizandrol B+OH（1） | C_23_H_28_O_8_ | 31.00 | 433.1857 | 433.1852[M+H]^+^  415.1746[M+H-H_2_O]^+^,  373.2742, 327.0881 |
| M5-2 | schizandrol B+OH（2） | C_23_H_28_O_8_ | 32.02 | 433.1857 | 415.1752[M+H-H_2_O]^+^,  385.1648[M+H-H_2_O-CH_2_O]^+^,  373.1286, 369.1707, 331.1188, 329.1018,  327.0864, 315.1234 |
| M5-3 | Schisandrin | C_24_H_32_O_7_ | 32.27 | 433.2221 | 415.2129[M+H-H_2_O]^+^，  384.1941[M+H-H_2_O-OCH_3_]^+^，  369.1706[M+H-H_2_O-OCH_3_-CH_3_]^+^ |
| M5-4 | Schizandrol B-CH2 | C_22_H_26_O_7_ | 29.70 | 403.1751 | 403.1736[M+H]^+^  385.1651[M+H-H_2_O]^+^,  329.1000[M+H-H_2_O-C_4_H_8_]^+^ |

**Supplementary Table S9** (Continued)

| Schisantherin A | | | | | |
| --- | --- | --- | --- | --- | --- |
| M6-0 | Schisantherin A | C_30_H_32_O_9_ | 36.94 | 537.2119 | 537.2105[M+H]^+^,  415.1764[M+H-C_6_H_5_COOH]^+^,  371.1501[M+H-C_6_H_5_COOH-C_2_H_4_O]^+^,  340.1316[M+H-C_6_H_5_COOH-C_2_H_4_O-OCH_3_]^+^,  325.1074[M+H-C_6_H_5_COOH-C_2_H_4_O-OCH_3_-CH_3_]^+^,  356.1271[M+H-C_6_H_5_COOH-C_2_H_4_O-CH_3_]^+^ |
| Schisandrin A | | | | | |
| M7-0 | Schisandrin A | C_24_H_32_O_6_ | 39.41 | 417.2272 | 417.2281[M+H]^+^,402.2052,  386.2089[M+H-OCH_3_]^+^,  316.1314[M+H-C_5_H_10_-OCH_3_]^+^,  301.1072[M+H-C_5_H_10_-OCH_3_-CH_3_]^+^ |
| M7-1 | Schisandrin A-O-CH4-CH2（1） | C_22_H_26_O_7_ | 19.52 | 403.1751 | 403.1744[M+H]^+^,  371.1507[M+H-H_2_O-CH_2_]^+^, 345.1324, 313.1088, 281.0814 |
| M7-2 | Schisandrin A-O-CH4-CH2（2） | C_22_H_26_O_7_ | 19.96 | 403.1751 | 403.1763[M+H]^+^, 345.1344, 313.1084, 281.0801,  253.0870 |
| M7-3 | Schisandrin A-C2H5+COOH | C_23_H_28_O_8_ | 25.44 | 433.1857 | 433.1877[M+H]^+^,  415.1714[M+H-H_2_O]^+^, |
| M7-4 | Schisandrin A-OCH2-4H+2O | C_23_H_26_O_7_ | 32.02 | 415.1751 | 415.1752[M+H]^+^,  397.1638[M+H-H_2_O]^+^,  385.1648[M+H-CH_2_O]^+^,  371.1503[M+H-CH_2_O-CH_2_]^+^,  356.1252,342.1103, 325.1078, 315.1234 |
| M7-5 | Schisandrin | C_24_H_32_O_7_ | 32.32 | 433.2221 | 433.2218[M+H]^+^，  415.2143[M+H-H_2_O]^+^，  384.1951[M+H-H_2_O-OCH_3_]^+^，  369.1704[M+H-H_2_O-OCH_3_-CH_3_]^+^ |

**Supplementary Table S9** (Continued)

| M7-6 | Schisantherin A | C_30_H_32_O_9_ | 36.99 | 537.2119 | 537.2105[M+H]^+^,  415.1764[M+H-C_6_H_5_COOH]^+^,  371.1501[M+H-C_6_H_5_COOH-C_2_H_4_O]^+^,  340.1316[M+H-C_6_H_5_COOH-C_2_H_4_O-OCH_3_]^+^,  325.1074[M+H-C_6_H_5_COOH-C_2_H_4_O-OCH_3_-CH_3_]^+^,  356.1271[M+H-C_6_H_5_COOH-C_2_H_4_O-CH_3_]^+^ |
| --- | --- | --- | --- | --- | --- |
| M7-7 | Schisandrin B | C_23_H_28_O_6_ | 40.52 | 401.1959 | 401.1969[M+H]^+^，  386.1731[M+H-CH_3_]^+^，  331.1182[M+H-C_5_H_10_]^+^,  300.0993[M+H-C_5_H_10_-OCH_3_]^+^ |
| Schisandrin B | | | | | |
| M8-0 | Schisandrin B | C_23_H_28_O_6_ | 40.52 | 401.1959 | 401.1969[M+H]^+^，  386.1731[M+H-CH_3_]^+^，  331.1182[M+H-C_5_H_10_]^+^,  300.0993[M+H-C_5_H_10_-OCH_3_]^+^ |
| Schisandrin C | | | | | |
| M9-0 | Schisandrin C | C_22_H_24_O_6_ | 41.03 | 385.1646 | 385.1656[M+H]^+^,  355.1563[M+H-CH_2_O]^+^，  285.0767[M+H-C_5_H_10_-CH_2_O]^+^ |
| M9-1 | Schisandrin B | C_23_H_28_O_6_ | 40.52 | 401.1959 | 401.1969[M+H]^+^，  386.1731[M+H-CH_3_]^+^，  331.1182[M+H-C_5_H_10_]^+^,  300.0993[M+H-C_5_H_10_-OCH_3_]^+^ |
| Taurodeoxycholic acid | | | | | |
| M10-0 | Taurodeoxycholic acid | C_26_H_45_NO_6_S | 27.54 | 498.2884 | 498.2896[M-H]^-^ |
| glycohyodeoxycholic acid | | | | | |
| M11-0 | glycohyodeoxycholic acid | C_26_H_43_NO_5_ | 29.81 | 448.3057 | 448.3073[M-H]^-^,  386.3079[M-H-H_2_O-CO_2_]^-^, 74.0248 |

**Supplementary Table S10.** Enrichment analysis results of the signal pathway of Hugan tablets.

| Signal pathway | Number of key  targets | Targets contained |
| --- | --- | --- |
| Pathways in cancer | 8 | CASP3, AKT1, HSP90AA1, HIF1A, EGFR, MAPK3, JUN, STAT3 |
| Hepatitis B | 6 | SRC, TNF, CASP3, MAPK3, JUN, STAT3 |
| Prostate cancer | 4 | EGFR, AKT1, MAPK3, HSP90AA1 |
| ProteoglASHans in cancer | 8 | SRC, HIF1A, TNF, EGFR, CASP3, AKT1, MAPK3, STAT3, |
| Lipid and atherosclerosis | 8 | SRC, TNF, CASP3, AKT1, MAPK3, JUN, HSP90AA1, STAT3 |
| AGE-RAGE signaling pathway in diabetic complications | 6 | TNF, CASP3, AKT1, MAPK3, JUN, STAT3 |
| Kaposi sarcoma-associated herpesvirus infection | 7 | SRC, HIF1A, CASP3, AKT1, MAPK3, JUN, STAT3 |
| EGFR tyrosine kinase inhibitor resistance | 5 | SRC, STAT3, EGFR, AKT1, MAPK3 |
| Pancreatic cancer | 4 | STAT3, EGFR, AKT1, MAPK3 |
| PI3K-Akt signaling pathway | 4 | EGFR, AKT1, MAPK3, HSP90AA1, |
| Human cytomegalovirus infection | 7 | SRC, TNF, EGFR, CASP3, AKT1, MAPK3, STAT3 |
| Endocrine resistance | 5 | SRC, EGFR, AKT1, MAPK3, JUN, |
| Central carbon metabolism in cancer | 4 | HIF1A, EGFR, AKT1, MAPK3 |
| BRagas disease | 4 | JUN, TNF, AKT1, MAPK3 |
| FoxO signaling pathway | 4 | EGFR, AKT1, MAPK3, STAT3 |
| TNF signaling pathway | 5 | JUN, TNF, CASP3, AKT1, MAPK3 |
| MAPK signaling pathway | 6 | TNF, EGFR, CASP3, AKT1, MAPK3, JUN, |
| HIF-1 signaling pathway | 5 | STAT3, HIF1A, EGFR, AKT1, MAPK3 |
| VEGF signaling pathway | 3 | SRC, AKT1, MAPK3 |
| Human immunodeficiency virus 1 infection | 5 | TNF, CASP3, AKT1, MAPK3, JUN |

**Reagents and materials**

Chemical reference substances, including apigenin8-C-glucoside (batach No. Y28N10H104438), guanosine (batach No. AJ0609NA14), saikosaponin b2 (SS2, batach No. Z08A8L33358) and glycochenodeoxycholic acid (batach No. Y29M9K57235), were bought from Yuanye Biotechnology Co., Ltd. (Shanghai, China). The reference standards, namely, schizandrol B (SZDB, batach No. 08S-CYO-55-3), schisandrin C (SSDC, batach No. 17J-BKD-99-7) as well as glycohyodeoxycholic acid (GA, batch No. 27D-SSD-11-9), were supplied via Tianjin Yifang Technology Co., Ltd. (Tianjin, China), and schisanhenol (batch No. PRF8041142) was from Chengdu Puruifa Technology Development Co., Ltd. (Chengdu, China). The other reference standards, containing taurodeoxycholic acid (TA, batch No. 111943-201802), schisandrin B (SSDB, batch No. 110765-201813), schisandrin (SSD, batch No. 110857-201815), chlorogenic acid (CA, batch No. 110753-201817), (R, S)-goitrin (batch No. 111753-202007), schisandrin A (SSDA, batch No. 110764-201915), rutinum (batch No. 100080-201811), scoparone (batch No. 111511-201704), schisantherin A (SSTA, batach No.111529-201706 ), hyperoside (batch No. 111521-201809) and quercetin (batch No. 100081-201610) were purchased from National Institutes for Food and Drug Control (Beijing, China). The purity of all reference standards was above 98%. Fifteen batches of HGT (number S1-S15, batch No. 202103150 ~ 202103164) and six herbs, named BR, ASH, PFS, SCF as well as MB, were provided by Heilongjiang Sunflower Pharmaceutical Co., Ltd. (Heilongjiang China). LC-MS grade acetonitrile, LC-MS grade methanol, LC-MS grade formic acid, as well as both methanol and acetonitrile of HPLC grade were purchased from Thermo Fisher Scientific Co., Ltd. (Waltham, USA). Analytical pure methanol and phosphoric acid were obtained from Tianjin Damao Chemical Reagent Factory (Tianjin, China). Distilled water was from Quchenshi Co., Ltd. (Hong Kong, China).

**Quantitative analysis and effectiveness evaluation of specific metabolites**

In CNKI, PubMed and Web of Science databases, “11 plant metabolites with measurability and specificity”, “liver fibrosis”, “alcoholic fatty liver”, “hepatitis”, “drug-induced liver injury’, “liver cancer” and “fatty liver” were used as keywords for literature mining. Therefore, the effectiveness of these 11 metabolites in the treatment of liver disease was evaluated.

The literature mining results were presented in Supplementary Figure S9. For examples, SS2 can inhibit endoplasmic reticulum stress signal pathway, the expression of nuclear factor-κB (NF-κB) protein, up-regulate Sirt-6 protein expression, and reduce the levels of alanine aminotransferase (ALT), aspartate aminotransferase (AST), TNF-α, IL-1β and IL-6 in serum to attenuate LPS/Gal N-induced acute liver injury in vivo (You et al., 2019; Lv et al., 2019). CA is a phenolic compound with anti-inflammatory and antioxidant activities, and is considered to be the major bioactive metabolite in ASH (Sun et al., 2013). Moreover, pharmacological studies have proved CA can ameliorate liver fibrosis in CCl4-injected rats by inhibiting oxidative stress and enhancing antioxidant defense via the Nrf2 pathway (Shi et al., 2013; Shi et al., 2016). SSDB has been proved to have in vivo efficacy against drug-induced liver injury, liver fibrosis and non-alcoholic liver disease (Li et al., 2014; Ma et al., 2022; Wang et al., 2022). Modern pharmacological research has demonstrated that most of the biological actions and pharmacological effects of SCF can be attributed to lignan constituents, which can lower the serum glutamate-pyruvate transaminase level, and show antioxidative, antitumor-promoting, and anti-HIV effects (Lu and Chen, 2009). SSD, SZDB, SSTA, SSDA and SSDC can prevent APAP-induced liver injury and chronic liver injury by inhibiting GSH depletion, down-regulating the level of ALT, AST, alkaline phosphatase and malonic dialdehyde, and suppressing CYP-mediated APAP metabolic activation (Jiang et al., 2015; Yan et al., 2009).

**Reference：**

Jiang, Y.M., Fan, X.M., Wang, Y., Tan, H.S., Chen, P., Zeng, H., et al. (2015). Hepato-protective effects of six schisandra lignans on acetaminophen-induced liver injury are partially associated with the inhibition of CYP-mediated bioactivation. *Chem. Biol. Interact.* 231, 83-89. doi: 10.1016/j.cbi.2015.02.022.

Li, L.B., Zhang, T.R., Zhou, L., Zhou, L., Xing, G.H., Chen, Y.H., et al. (2014). Schisandrin B attenuates acetaminophen-induced hepatic injury through heat-shock protein 27 and 70 in mice. *J Gastroen and Hepatol.* 29(3), 640-647. doi: 10.1111/jgh.12425.

Lu, Y., and Chen, D.F. (2009). Analysis of *Schisandra chinensi*s and *Schisandra sphenanthera*. J Chromatogr A. 1216(11), 1980-1990. doi: 10.1016/j.chroma.2008.09.070.

Lv, X.Z., Li. R.F., Gao, Z.H., Wang. H.W., Li, S.Q., Wang, J. G. (2019). Saikosaponin-b2 alleviates CCl_4_-induced acute liver injury in mice by inhibiting endoplasmic reticulum stress signal pathway. *Chin J Pharmacol Toxcicol*. 33 (2). 109-115. doi: 10.3867/j.issn.1000-3002.2019.02.004.

Ma, R.J., Zhan, Y.K., Zhang, Y.M., Wu, L.A., Wang, X., and Guo, M. (2022). Schisandrin B ameliorates non-alcoholic liver disease through anti-inflammation activation in diabetic mice. *Drug Develop Res.* 83(3), 735-744. doi: 10.1002/ddr.21905.

Ren, L.L., Yao, D. Y., Ren, Ying., Li, X.W. (2022). Study on quality control of Schisandra Transaminase reduction capsule. *Anhui Med Pharm J*. 26(4)：676-679. doi：10.3969/j.issn.1009-6469.2022.04.008.

Shi, H., Dong, L., Jiang, J., Zhao, J., Zhao, G., Dang, X., et al. (2013). Chlorogenic acid reduces liver inflammation and fibrosis through inhibition of toll-like receptor 4 signaling pathway. *Toxicology* 303, 107-114. doi: 10.1016/j.tox.2012.10.025.

Shi, H.T., Shi, A., Dong, L., Lu, X.L., Wang, Y., Zhao, J.H., et al. (2016). Chlorogenic acid protects against liver fibrosis in vivo and in vitro through inhibition of oxidative stress. *Clin Nutr*. 35(6), 1366-1373. doi: 10.1016/j.clnu.2016.03.002.

Sun, H., Zhang, A., Yan, G., Han, Y., Sun, W., Ye, Y., et al. (2013). Proteomics study on the hepatoprotective effects of traditional Chinese medicine formulae Yin-Chen-Hao-Tang by a combination of two-dimensional polyacrylamide gel electrophoresis and matrix-assisted laser desorption/ionization-time of flight mass spectrometry. *J Pharm Biomed Anal*. 75, 173-179. doi: 10.1016/j.jpba.2012.11.025.

Wang, H.Q., Wan, Z., Zhang, Q.Q., Su, T., Yu, D., Wang, F., et al. (2022). Schisandrin B targets cannabinoid 2 receptor in Kupffer cell to ameliorate CCl4-induced liver fibrosis by suppressing NF-kappa B and p38 MAPK pathway. *Phytomedicine* 98. doi: 10.1016/j.phymed.2022.153960.

Yan, F., Zhang, Q.Y., Jiao, L., Han, T., Zhang, H., Qin, L.P., et al. (2009). Synergistic hepatoprotective effect of Schisandrae lignans with Astragalus polysaccharides on chronic liver injury in rats. *Phytomedicine* 16(9), 805-813. doi: 10.1016/j.phymed.2009.02.004.

You, M., Li, R.F., Gao, Z.H., Li, Y.Y., Liu, W.Y., Wang, J.G., et al. (2019). Effects of saikosaponin b2 on inflammation and energy metabolism in mice with acute liver injury induced by LPS/GalN. *Chin J Chin Mater Med*. 44(14), 2966-2971. doi: 10.19540/j.cnki.cjcmm.20190521.415.
